# Linking transcriptional dynamics of CH_4_-cycling grassland soil microbiomes to seasonal gas fluxes

**DOI:** 10.1038/s41396-022-01229-4

**Published:** 2022-04-06

**Authors:** Jana Täumer, Sven Marhan, Verena Groß, Corinna Jensen, Andreas W. Kuss, Steffen Kolb, Tim Urich

**Affiliations:** 1 Institute of Microbiology, Center for Functional Genomics of Microbes, University of Greifswald, Greifswald, Germany; 2 Institute of Soil Science and Land Evaluation, Soil Biology Department, University of Hohenheim, Stuttgart, Germany; 3 Human Molecular Genetics Group, Department of Functional Genomics, University Medicine Greifswald, Greifswald, Germany; 4 RA Landscape Functioning, Leibniz Centre for Agricultural Landscape Research (ZALF), Müncheberg, Germany; 5 Thaer Institute, Faculty of Life Sciences, Humboldt University of Berlin, Berlin, Germany

**Keywords:** Soil microbiology, Transcriptomics, Microbial ecology, Next-generation sequencing

## Abstract

Soil CH_4_ fluxes are driven by CH_4_-producing and -consuming microorganisms that determine whether soils are sources or sinks of this potent greenhouse gas. To date, a comprehensive understanding of underlying microbiome dynamics has rarely been obtained in situ. Using quantitative metatranscriptomics, we aimed to link CH_4_-cycling microbiomes to net surface CH_4_ fluxes throughout a year in two grassland soils. CH_4_ fluxes were highly dynamic: both soils were net CH_4_ sources in autumn and winter and sinks in spring and summer, respectively. Correspondingly, methanogen mRNA abundances per gram soil correlated well with CH_4_ fluxes. Methanotroph to methanogen mRNA ratios were higher in spring and summer, when the soils acted as net CH_4_ sinks. CH_4_ uptake was associated with an increased proportion of USCα and γ *pmoA* and *pmoA*2 transcripts. We assume that methanogen transcript abundance may be useful to approximate changes in net surface CH_4_ emissions from grassland soils. High methanotroph to methanogen ratios would indicate CH_4_ sink properties. Our study links for the first time the seasonal transcriptional dynamics of CH_4_-cycling soil microbiomes to gas fluxes in situ. It suggests mRNA transcript abundances as promising indicators of dynamic ecosystem-level processes.

## Introduction

CH_4_ is a powerful greenhouse gas [1]. Between 41% and 53% of global CH_4_ emissions derive from aquatic systems. Therein freshwater wetlands are the largest single source, emitting about 138–165 Tg CH_4_ yr^−1^ [2, 3]. Since 1700, between 54% and 57% of the wetlands were lost due to drainage to gain agricultural land, such as grasslands [4, 5]. Drainage lowers the water table, altering water content and oxygen availability. These altered soil physical conditions, in turn, substantially affect the soil microbiota and activity and thus the soils’ greenhouse gas fluxes [6, 7]. Drained former wetlands are a large source of CO_2_ but can also emit substantial amounts of CH_4_, depending on their dynamic hydrological status throughout the year [4, 5].

More than two-thirds of global CH_4_ emissions derive from microbial production [8]. CH_4_-producing microbes (i.e., methanogens) are mostly anaerobic Archaea that inhabit anoxic environments [8, 9]. Four types of methanogens can be characterized according to their substrate specificity. Acetoclastic methanogens utilize acetate, hydrogenotrophic methanogens utilize H_2_/CO_2_ and formate, and methylotrophic methanogens utilize methanol/methylamines to form CH_4_ [9]. Recently, methoxydotrophic methanogens that utilize methoxylated aromatic compounds were proposed as a novel methanogenic group [10, 11]. In soils, acetoclastic and hydrogenotrophic methanogens are considered the predominant sources of CH_4_ [9, 12]. However, recent research indicates that methanogenesis from methylated compounds also contributes to CH_4_ emissions from soils and wetlands [13, 14].

Up to 90% of CH_4_ produced in oxygen-limited soils can be mitigated through oxidation by aerobic methane-oxidizing Bacteria (MOB) within the lineages *Alphaproteobacteria*, *Gammaproteobacteria*, and *Verrucomicrobia* [15–17]. CH_4_ oxidation can also be conducted anaerobically by Bacteria of the NC10 phylum and Archaea in the ANME group that couple oxidation of CH_4_ to the reduction of other electron acceptors such as nitrite (NC10), nitrate (ANME-2d), or ferric iron [18–20]. Aerobic methanotrophs are considered the main oxidizers in wetland soils since alternative electron acceptors favoring anaerobic methanotrophs are often scarce in wetland soils. Tracing stable isotopes and radioisotopes can link CH_4_ consumption to active methanotrophs [21–25]. For instance, incubating soil cores with ^13^C-CH_4_ identified γ-proteobacterial subgroups as the main active methanotrophs in a riparian floodplain [22]. Additionally, methanotrophs provide the only known biological sink for atmospheric CH_4_ [26]. However, it is not fully understood which microorganisms oxidize CH_4_ at atmospheric concentrations in soils. Bacteria of upland soil clusters (USC)α and USCγ have been identified as likely important atmospheric MOBs in upland soils [15, 27–29], while well-known methanotrophic lineages may also oxidize atmospheric CH_4_ in anoxic paddy soils [30]. A study using stable-isotope labeled CH_4_ identified type II methanotrophs related to *Methylocapsa acidophila* active in grassland and forest soils at low CH_4_ concentrations [25].

Presumably, the combined net activities of methanogens and methanotrophs determine whether wetland soils act as net sources or sinks for CH_4_ [31]. However, linking CH_4_-cycling microbiome dynamics of soils in situ to CH_4_ fluxes, especially at the transcriptional level, has rarely been achieved [32]. DNA- and RNA-based meta-omics techniques have provided insight into the microbiome compositions of soils. However, DNA is long-term stable; extracted soil DNA may therefore partially originate from persistent extracellular DNA of dead organisms [33, 34]. In contrast, ribosomal RNA (rRNA) acts as a proxy for ribosomes. Even though dormant cells can contain high loads of ribosomes [35, 36], RNA-SIP studies [37, 38] indicate that approximately 94% of microbial taxa in soil are active and synthesize new rRNA [39]. Still, rRNA content does not necessarily reflect the gene expression. Hence, although rRNA is a good proxy for potential active soil microbiome, it may not relate well to ecosystem processes. The simultaneous sequencing of mRNA and rRNA potentially can overcome this issue [40] because messenger RNA (mRNA), can serve as a proxy for transcriptional activity. Other metatranscriptome studies indicate that mRNA is more responsive to environmental factors than rRNA [41, 42]. For instance, methanogen-related mRNA, but not SSU rRNA, decreased in soil microcosms exposed to drought [43]. The relationship between the abundances of rRNA and mRNA of CH_4_-cycling microbes and CH_4_ fluxes has not been studied in situ. We thus aim to explore differences between small subunit (SSU) rRNA and mRNA transcripts of the CH_4_-cycling microbiomes and their links to gas fluxes.

Another drawback of meta-omics techniques is that they usually yield only relative abundances. However, the relationship between absolute abundances and relative abundances is not predictable [44]. It is thus challenging to relate ecosystem processes to relative abundances. Studies have applied absolute quantification for metatranscriptomes in marine microbiomes [45, 46]. Recently, a quantification approach that uses total RNA to infer absolute from relative abundance has been developed for metatranscriptomics [47].

In this study, we aimed to link transcriptional dynamics of CH_4_-cycling microbiomes to CH_4_ fluxes in two grassland soils. These soils were wetlands in the past but have been drained for agricultural use several decades ago. We used quantitative metatranscriptomics to analyze ribosomal rRNA and mRNA [40, 47] of 60 soil samples taken from different soil depths during autumn, winter, spring, and summer. In addition, we measured CH_4_ and CO_2_ net surface fluxes from the two sites. We aimed to (a) evaluate the RNA content of the soils as a marker for microbial activity, (b) examine the CH_4_ fluxes of the two in grasslands throughout a year, (c) study the composition and abundance of SSU rRNA and mRNA transcripts of CH_4_-cycling microbes, and (d) link microbiome composition of CH_4_-cycling organisms to net surface CH_4_ fluxes across seasons.

## Materials and methods

### Site description

The experiment was conducted in the framework of the Biodiversity Exploratories project for long-term functional ecosystem research [48]. Samples were taken at two grassland sites (LI and HI) located in the Biosphere Reserve „Schorfheide-Chorin“ (Supplementary Table S1). Both sites are drained peatlands with a histosolic soil type (according to WRB 2015 [49]). The upper 30 cm of the peat soils was highly degraded. The two sites differ in the intensity of grassland management; the low land-use intensity site (LI) was mowed once or twice a year, while the high land-use intensity site (HI) was grazed by cows (400–700 livestock units * grazed days ha^-1^ y^-1^) and additionally mowed sometimes once a year. Vegetation on LI was dominated by *Poa trivialis* (60%) and *Alopecurus pratensis* (25%); vegetation on HI was dominated by *Poa pratensis* aggr. (32 %), *Trifolium repens* (15%) and *Agrostis stolonifera* (10%).

### Soil Sampling

On each site, an area of 1 m × 7 m was sampled at all four seasons: autumn (11/09/2017), winter (03/08/2018), spring (05/30/2018), and summer (09/13/2018). At each sampling date, three spatial replicate samples were taken between 12:00 and 13:00 at each site from the upper 10 cm and the 20–30 cm layer. Each soil sample was a mixture of the respective soil layer from three soil cores, taken close to each other (5–10 cm). The replicates were located at least 1 m apart from each other. At each seasonal sampling, the replicates were taken at least 1 m apart from replicates taken during the previous sampling campaigns. In spring, additional samples were taken at sunrise (05:00) and sunset (21:30), but only at the HI site. Samples for RNA, ammonium (NH_4_^+^), and nitrate (NO_3_^−^) extraction were immediately frozen at −80 °C and subsequently stored as follows: RNA: −80 °C, NH_4_^+^, and NO_3_^−^ −20 °C. Samples for determination of C_mic_, N_mic_, pH, and soil water content were transported on ice and subsequently stored at −20 °C. Redox potentials were measured with Mansfeld redox electrodes with an Ag/AgCl-reference electrode and a handheld ORP-meter GMH3531 (ecoTech, Bonn, Germany). For equilibration. the electrodes were placed in the soil 24 h before sampling. Redox potentials were measured at soil depths of 5 cm and 25 cm.

### Determination of soil properties

Gravimetric soil water content was determined by drying 3–6 g soil at 65 °C to constant weight. Soil pH was determined by mixing 10 g dried sieved soil with 25 ml 0.01 M CaCl_2_ solution; pH of the suspension was then measured with a glass electrode (pH Electrode LE438, Mettler Toledo, Columbus, OH, USA). For total carbon and total nitrogen, samples were sieved (< 2 mm) and air-dried, ground in a ball mill (RETSCH MM200, Retsch, Haan, Germany), and analyzed in an elemental analyzer (VarioMax, Hanau, Germany) at 1100 °C. Inorganic carbon was determined with the same elemental analyzer after the organic carbon had been removed by combustion of soil samples at 450 °C for 16 h. Organic carbon concentration was calculated as the difference between total carbon and inorganic carbon. Microbial biomass carbon (C_mic_) and nitrogen (N_mic_) were determined by the chloroform-fumigation-extraction method (CFE) [50]. For this, frozen soils were thawed (at 4 °C for 10 h), then 5 g field moist soils were fumigated with ethanol-free CHCl_3_ for 24 h in a desiccator. C and N were extracted with 40 ml 0.5 M K_2_SO_4_, shaken horizontally (30 min, 150 rpm), and centrifuged (30 min, 4400 g) to separate extract from the soil. Non-fumigated soil samples were treated identically. Aliquots of the extracts were dissolved (1:4 extract:deionized. H_2_O) and measured on a TOC/TN analyzer (Multi N/C 2100S, Analytik Jena AG, Jena, Germany). A kEC factor [51] and a kEN factor [52] were used to calculate C_mic_ and N_mic_, respectively. The organic C and N content determined from non-fumigated samples were used as a measure for the extractable C (EOC) and N (EN) which can be considered as microbially available resource in soil [53]. Mineral nitrogen in the forms of ammonium (NH_4_^+^) and nitrate (NO_3_^−^) was determined in the non-fumigated, non-diluted extracts with an AutoAnalyzer 3 (Bran & Luebbe, Norderstedt, Germany).

### Gas fluxes

On each sampling date, gas emissions were measured with four closed chambers per site. With each chamber, the measurements were repeated four to six times per day and site, resulting in 15–24 net surface rate measurements. Excessive vegetation was removed before pressing the stainless steel chambers (*A* = 150 cm^2^, *V* = 1800 ml) into the soil [54]. The chambers had a sharp-edged bottom, which allowed the installation in the organic soils without compacting the soil. Gas samples (12 ml) were taken with syringes from the headspace immediately, 20, 40, and 60 min after closing the chambers via a three-way stopcock, and transferred into pre-evacuated exetainers (5.9 ml, Labco Lt, UK). Gas concentrations were measured on an Agilent 7890 gas chromatograph equipped with a flame ionization detector (for CH_4_) coupled with a methanizer (for CO_2_) (Agilent Technologies Inc., Santa Clara, CA, USA). Gas flux rates were calculated by the slope of the regression line of a linear regression of the gas concentration against time [27].

### RNA extraction, library preparation, and sequencing

Total nucleic acids were extracted using a phenol/chloroform/isoamylalcohol protocol [40]. The extracts were subsequently treated with DNase to remove DNA (DNase I, Zymo Research, Freiburg, Germany). RNA concentrations were measured with the Qubit RNA HS Assay Kit (Qubit3.0 Fluorometer, Invitrogen, Waltham, MA, USA.). RNA extracts were cleaned with the MEGAclear kit (Thermo Fisher Scientific, Waltham, MA, USA); the quality of the RNA was verified by agarose gel electrophoresis and bioanalyzer (2100 Bioanalyzer, Agilent, Santa Clara CA, USA). We enriched the mRNA fraction and diluted inhibitory substances in the RNA extracts using the MessageAmp II-Bacteria RNA Amplification Kit (Thermo Fisher Scientific, MA, USA, input: 12.5 ng RNA). This method was previously validated for the preparation of metatranscriptomes [55]. Sequencing libraries were prepared with NEBNext Ultra II RNA Library Prep Kit for Illumina (New England Biolabs, Ipswich, MA, USA; input 60 ng). Manufacturer’s instructions were followed except for Step 4, where fragmentation time was adjusted to 3 min and a size selection step with HighPrep PCR beads (MagBio Genomics Inc., Gaithersburg, USA) was introduced (desired insert size 250 bp). Libraries were paired-end sequenced with a NextSeq 550 System using the NextSeq 500/550 High Output Kit v2.5 (300 Cycles) (Illumina, San Diego, CA, USA).

### Bioinformatic processing and statistics

Reverse and forward sequences were overlapped with a minimum overlap of 10 or 5 bp with FLASH [56]. The sequences were filtered to a minimum mean quality score of 25 with PrinseqLite [57]. Sequences were then sorted into SSU rRNA, LSU rRNA, and non-rRNA fractions with SortMeRNA [58]. The SSU rRNA fraction was randomly subsampled to 200000 sequences with USEARCH [59]. Sequences were taxonomically classified against the SilvaMod128 databases [60] with BlastN [61] using a lowest common ancestor (LCA) algorithm in MEGAN (min score 155; top percent 2.0; min support 1 [62]). The non-rRNA fraction was aligned against the NCBI_nr database (retrieved 12/03/2020) with Diamond [63]. The sequences were taxonomically and functionally aligned with LCA in MEGAN (2011, min score 155; top percent 4; min support 1 [62]). Absolute abundances were calculated from read counts according to Söllinger et al. [47]. This calculation integrates the relative read abundance obtained from metatranscriptomics with the amount of mRNA and SSU rRNA extracted from the soil, respectively, and the average number of transcripts per µg RNA. At mRNA level, methanogenesis transcripts refer to sequences assigned to the SEED category “methanogenesis”. Methanotrophy transcripts refer to sequences assigned to the SEED category “Particulate methane monooxygenase (pMMO)”. To classify *pmoA* sequences, the non-rRNA fraction was searched against a *pmoA* database [64] and taxonomically classified with MEGAN as described in reference [64]. To assess the transcriptional activity of CH_4_-cycling microbes throughout the years, we binned mRNAs taxonomically classified as methanogens (Euryarchaeota) and alpha and gammaproteobacterial methanotrophs, respectively to then analyze the functionally assigned mRNAs using SEED and KEGG.

Statistical analyses were performed in R [65]. Distance-based redundancy analysis was performed on the Bray–Curtis dissimilarity matrix read counts of the 60 samples (function “dbrda” in the vegan package [66]). Counts were Hellinger-transformed beforehand. We tested the following parameters: site (HI; LI), depth (“0–10 cm”, “20–30 cm”), season (“autumn”,”winter”, “spring”, “summer”), temperature, water content, nitrite, and nitrate. Continuous variables were z-scaled. The difference of transcript abundances and the ratio of methanotrophs to methanogens between seasons at one and the same site was assessed by ANOVA and subsequent post-hoc Tukey’s test, resulting in adjusted *p*-values. We used the arithmetic mean of methanogenesis and methanotroph transcript abundances from the upper (0–10 cm) and the lower (20–30 cm) of one sample. Significant differences between seasons were identified with the R package “multcompView” with *p*-adjusted <0.05 [67].

## Results and discussion

### Highly dynamic CH_4_ fluxes across the year

**Fig. 1:**
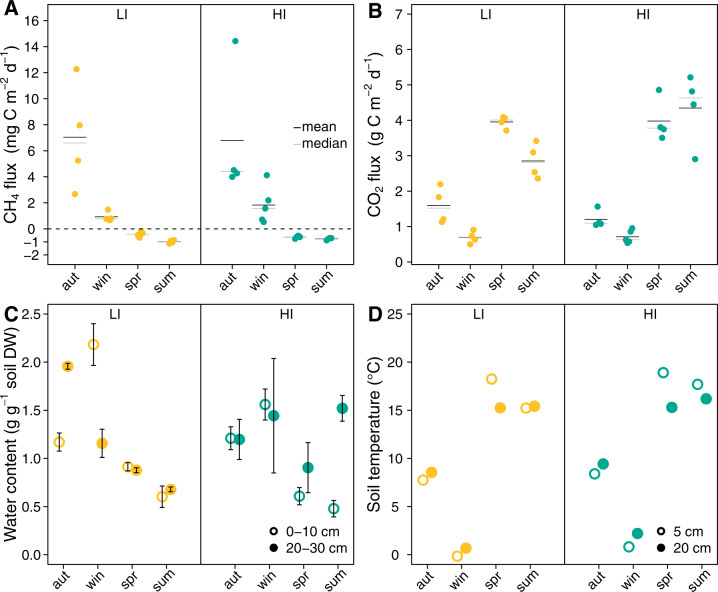
Net surface gas fluxes, soil temperature, and water content. Gas fluxes of CH_4_ (**A**), CO_2_ (**B**), gravimetric soil water content (**C**), and temperature (**D**) in the soils of the grassland site with low (yellow, LI) and high (turquoise, HI) land-use intensity in autumn (aut) 2017 and winter (win), spring (spr), and summer (sum) 2018. In **A** and **B**, one point shows the average of 4–6 repeated measurements of one chamber across one day; the mean and median are indicated with a black and gray line, respectively. In **C**, one point represents the mean and standard deviation of three replicates taken at noon, *n* = 3. In **D**, points represent the temperature measured at 12:00 in 5 cm and 20 cm soil depth, respectively.

We measured net surface fluxes from two grasslands across one day during autumn, winter, spring, and summer to assess their seasonal variation, in particular CH_4_. Daytime did not affect CH_4_ emissions (Supplementary Fig. 1A). In contrast, CH_4_ and CO_2_ fluxes were highly dynamic throughout the year (Fig. 1). While the soils emitted CH_4_ in autumn and winter (7.0 and 6.9 mg C m^−2^d^−2^, in autumn in LI and HI, respectively), they took up CH_4_ in spring and summer (−0.9 and −0.8 mg C m^−2^d^−2^, in summer in LI and HI, respectively) (Fig. 1A). CO_2_ fluxes showed an opposite trend, with higher CO_2_ emissions in spring and summer than in autumn and winter (Fig. 1B). The opposing trends of CO_2_ and CH_4_ fluxes reflected the changes in soil physicochemical properties across the year (Fig. 1C, D, Supplementary Table S2). Especially water content and temperature were likely the key factors in regulating gas turnover. High water content and low redox potentials in autumn and winter (Fig. 1C, Supplementary Table S2) likely favored anaerobic microbial processes, such as methanogenesis, while at the same time hampering aerobic microbial processes such as respiration (Supplementary Fig. 2). Low temperatures in winter likely resulted in smaller CH_4_ fluxes compared to autumn (Fig. 1A, D). In spring and summer, soils had lower water content and positive redox potential favoring aerobic over anaerobic degradation processes. Generally, mean CO_2_ net surface emissions were about 1.5 times higher than IPCC default emission factors [68, 69]. Our observed higher emissions may have been due to the degraded peat at the studied site. Soils with highly disturbed peat have been reported to have higher CO_2_ emissions than less degraded peat soils [70]. Next to soil water content, also temperature may have caused relatively high CO_2_ emissions as spring and summer 2018 were dry and hot compared to the long-term average. High temperatures increase organic matter decomposition and CO_2_ emissions [71, 72].

Net surface CH_4_ emissions rates in autumn and winter were lower compared to IPCC default emission factors [68]. However, we measured emissions at only four days and may have not accounted for high emissions after heavy rainfall events. Net CH_4_ uptake rates in spring and summer were in the range of other herbaceous and temperate ecosystems (0.36 and 0.47 ± 0.63 mg C m^−2^d^−1^) [73, 74] and higher than in pastures (mean 0.05 mg C m^−2^d^−1^) [74]. The beginning drought in 2018 caused low soil water content (Supplementary Table S2), favoring CH_4_ oxidation. The soil water content of the upper layer was mostly within the optimal range for atmospheric CH_4_ oxidation [75].

Our results underscore the high temporal variability of greenhouse gas emissions from temperate drained peatlands and their dependence on dynamic soil physicochemical properties, like temperature and soil moisture, which are themselves linked to seasons. Moreover, depending on the time of the year and conditions in the soil such sites can be net sinks for CH_4_ as well as net sources. This versatility regarding CH_4_ sink and source functions requires further long-term monitoring of such groundwater-impacted and organic-rich drained grassland soils in postglacial landscapes to ensure proper consideration in global budgets.

### Linking metatranscriptomics and microbial biomass

**Fig. 2:**
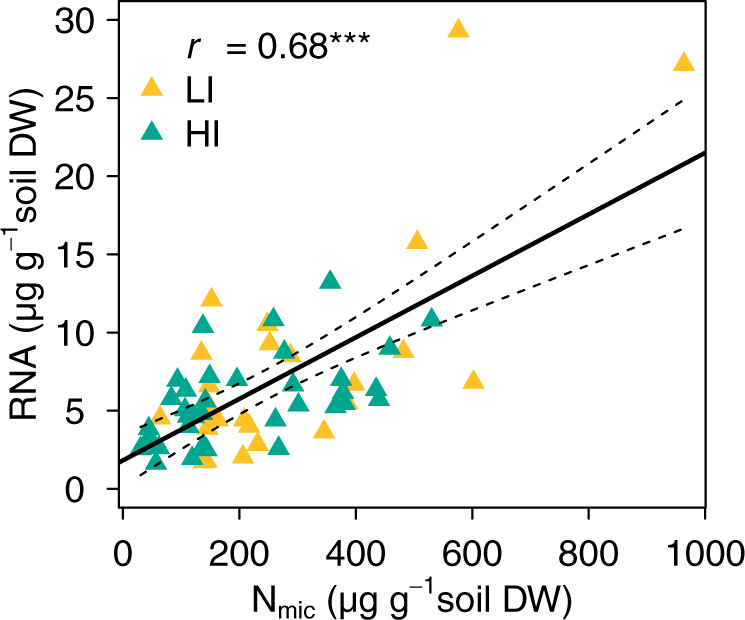
RNA and microbial biomass nitrogen content. Correlation between RNA content and microbial nitrogen content (N_mic_) per g soil dry weight (DW) in the soils of the grassland sites with low (LI, yellow) and high (HI, turquoise) land-use intensity. Linear regression RNA = 1.8182 + 0.0197 N_mic_, df = 58 (dashed lines show 95% CI). The “*r*” denotes the Pearson correlation coefficient. Significance codes: ***p < 0.001, *n* = 60.

We quantified soil total RNA content to examine if it reflects microbial biomass in the soils. Total RNA and N_mic_ and C_mic_ were determined from 60 top- and subsoil samples. They exhibited similar dynamics across seasons. Overall, total RNA per gram soil was positively correlated with both N_mic_ and C_mic_ (*r*_Nmic_ = 0.68, *r*_Cmic_ = 0.54, *p* < 0.001, Fig. 2, Supplementary Fig. 3). The RNA content correlated better with N_mic_, than with C_mic_, likely due to the high nitrogen content of the RNA. This finding supports the validity of RNA as a proxy for living microorganisms and the use of RNA content to infer transcript abundances per gram soil from relative transcript abundances obtained in metatranscriptomics [47]. Through this quantitative approach, one can overcome challenges typically associated with the interpretation of relative abundance data in ‘meta-omics’ datasets. A recent study used this quantitative approach and found that absolute transcript abundance correlated better to ecosystem processes than relative transcript frequencies [47].

### Spatial and seasonal dynamics in CH_4_-cycling (micro-)biomes

**Fig. 3:**
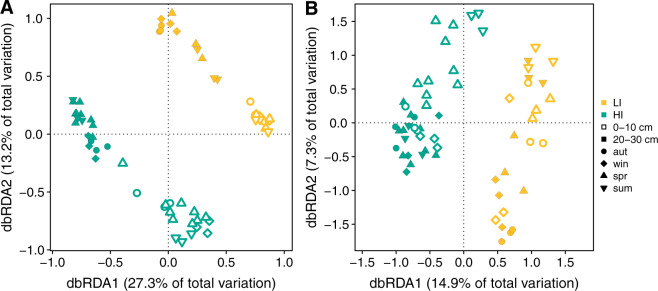
Soil (micro-)biome composition at the two grassland sites. Distance-based redundancy analysis (dbRDA) of the Bray–Curtis dissimilarity matrix of all 39,854 bacterial, archaeal and eukaryotic taxa (**A**) and the 287 CH_4_-cycling Archaea and Bacteria (**B**) in the soils of the grassland sites with low (LI, yellow) and high (HI, turquoise) land-use intensity from the upper (0–10 cm) and the deeper soil layer (20–30 cm) taken in autumn (aut) 2017 and winter (win), spring (spr) and summer (sum) 2018. Samples from autumn, winter, spring, and summer are depicted as circles, diamonds, upward-pointing triangles, and downward-pointing triangles, respectively.

High-throughput sequencing of metatranscriptomes yielded approximately 20 million paired-end reads per sample [76]. Three-domain analysis based on SSU rRNA reads revealed that the (micro-)biomes of the 60 samples were dominated by Bacteria, followed by eukaryotes and Archaea (Supplementary Tables S3 and S4, Supplementary Fig. 4). The community composition of all taxa in the soil samples exhibited a clear site- and depth-specific pattern (Fig. 3A), with site and depth explaining 20.0% and 19.6% of the variance, respectively (*p* < 0.001, Supplementary Table S5). Site-specific differences are likely attributed to site-specific soil properties, such as pH, texture, organic carbon, and nitrogen content, and land-use intensity (Supplementary Table S1). Depth is generally considered to be associated with differences in oxygen and nutrient availability. Eukaryotes were usually higher abundant in the upper soil layer, compared with the lower soil layer (Supplementary Fig. 4).

The composition of CH_4_-cycling microbes was also influenced by site, season, and depth (Fig. 3B). Site had the most explanatory power (14.0%, *p* < 0.001), but season, depth, and water content accounted for 6.5%, 5.7%, and 5.3% (*p* < 0.001) of the variance, respectively (Supplementary Table S6). Thus, the seasonal variability of the CH_4_ fluxes was accompanied by seasonal changes in CH_4_-cycling community composition. The seasonal effect likely resulted from varying precipitation, water table depth, and plant growth activity throughout the year. Especially the drought in spring and summer may have strongly affected the CH_4_-cycling microorganisms by lowering the soil water content. Oxygen diffusion into dry soils is much faster than into water-saturated soils, resulting in a higher O_2_ availability, which, in turn, is a fundamental factor shaping CH_4_-cycling community composition [77].

### Methanogen community composition and transcriptional activity

**Fig. 4:**
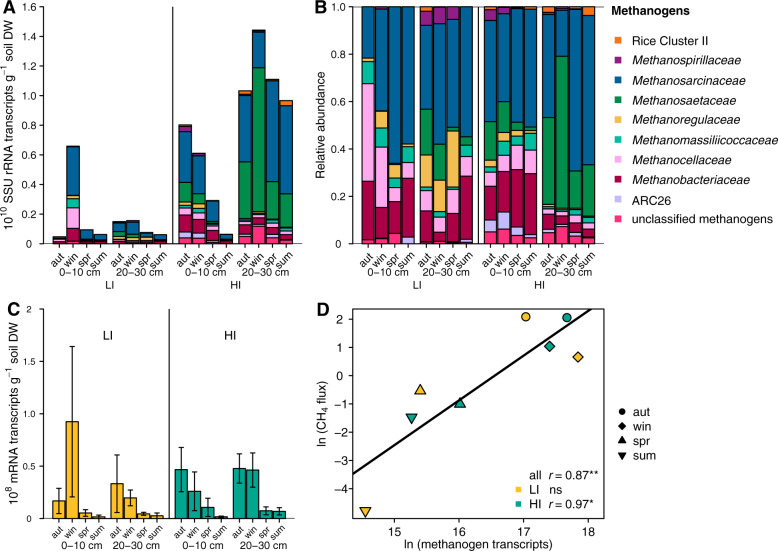
Methanogen SSU rRNA and mRNA abundances across seasons and depths. Absolute abundances (SSU rRNA transcripts g^−1^ soil DW) of methanogenic Archaea (**A**), the relative abundance of SSU rRNA transcripts belonging to methanogenic Archaea normalized to the total amount of SSU rRNA transcripts belonging to methanogenic Archaea (**B**), and transcript abundances (mRNA transcripts g^−1^ soil DW) of mRNA of methanogenesis pathways (**C**) in soils from the upper (0–10 cm) and the deeper soil layer (20–30 cm) of the grassland sites with low (LI, yellow) and high (HI, turquoise) land-use intensity taken in autumn (aut) 2017 and winter (win), spring (spr) and summer (sum) 2018. In **A**, **B**, and **C**, columns show means per season and depth of the upper (0–10 cm) and the deeper soil layer (20–30 cm) in LI and HI. “unclassified methanogens” contain methanogens unclassified at the class level and low abundance methanogenic groups. Bars represent the means of three replicates. In **C**, error bars represent the means and the standard deviations of three replicates. Linear correlation of absolute abundances of methanogenesis mRNA transcripts with CH_4_ fluxes (**D**). In **D**, points represent seasonal means across both depths; samples from autumn, winter, spring, and summer are depicted as circles, diamonds, upward-pointing triangles, and downward-pointing triangles, respectively. The “*r*” denotes the Pearson correlation coefficient. Significance codes: *p < 0.05, **p < 0.01, ns not significant. DW dry weight. We refer to Supplementary Fig. 5 and 6 showing the absolute and relative abundances of methanogen SSU rRNA in the individual samples, respectively.

We aimed to evaluate if SSU rRNA and mRNA abundances of CH_4_-cycling microbes reflected the seasonal changes in CH_4_ fluxes of the soils. For this purpose, we integrated the total RNA content and metatranscriptomes [47] to infer methanogen SSU rRNA and mRNA transcript abundances per gram soil (Fig. 4A, C). Generally, methanogen SSU rRNA abundances were higher in autumn and winter and the deeper soil layer, with abundances up to 1.4 ×10^10^ transcripts g^−1^ soil (Fig. 4A). Most methanogen families in the soils were class II methanogens, e.g., *Methanosarcinaceae*, *Methanosaetaceae* (now *Methanotrichaceae*) (Fig. 4B) which generally possess more antioxidant features than class I methanogens [78]. The predominance of class II methanogens likely reflected the dynamic water and redox status across seasons (Fig. 1C, Supplementary Table S2).

Methanogenesis mRNA transcripts were generally less abundant in spring and summer (0.21 and 0.43 * 10^7^ transcripts g^−1^ in summer in LI and HI, respectively) than in autumn and winter (5.6 and 3.6 * 10^7^ transcripts g^−1^in winter in LI and HI, respectively) (Fig. 4C). According to Tukey’s HSD test, methanogenesis transcript abundances were significantly lower (*p* < 0.05) in spring and summer compared to autumn and winter, in both LI and HI (Supplementary Tables S7 and 8). This drop in methanogenesis mRNA agrees with the cessation of CH_4_ emissions from the soils in spring in summer; both correlated significantly with each other (*r* = 0.87, *p* < 0.01, Fig. 4D). In contrast, the abundances of methanogen SSU rRNA transcripts and CH_4_ fluxes did not correlate significantly (Supplementary Fig. 7). Our results indicate that methanogenesis mRNA transcripts are better indicators of net CH_4_ fluxes than methanogen SSU rRNA transcripts (Fig. 4D, Supplementary Fig. 7). We thus underscore studies that have found mRNA more responsive to environmental factors than rRNA [41, 42].

We only sampled two sites and cannot make statistically assured statements about the influence of land-use intensity. Nevertheless, we observed some site-specific patterns. Methanogen SSU rRNA transcript abundances were higher in HI than in LI soils (Fig. 4A) despite similar methanogenesis mRNA transcript abundances (Fig. 4C). The taxonomic composition may influence the transcriptional activity of methanogenesis transcripts (Fig. 4B). The strictly acetoclastic *Methanosaetaceae* (*Methanothrix*) were more pronounced in HI than in LI (Fig. 4B). *Methanosaeta* have lower growth rates and can grow at lower acetate concentrations than the metabolically diverse *Methanosarcina* [79]. In turn, the share of hydrogenotrophic methanogens, such as *Methanocellaceae*, *Methanoregulaceae*, and *Methanobacteriaceae*, was higher in LI than in HI. The energy yield of hydrogenotrophic methanogenesis is larger than that of acetoclastic methanogenesis [9, 80]. The varying proportions of acetoclastic and hydrogenotrophic methanogens and lower acetate concentrations may explain lower transcriptional activity at HI compared to LI. Messenger RNA transcripts that were unambiguously attributed to a certain methanogenesis pathway, support that the share of acetoclastic mRNAs was lower in LI than in HI (Supplementary Fig. 8). However, large-scale studies, that include more sites would be needed to explore this effect further.

The consistent presence throughout the year of the obligate methylotrophic *Methanomassiliicoccales* (up to 14% of the methanogen SSU rRNA in the topsoils, Fig. 4B) points to methylated compounds as additional substrates for methanogenesis in both sites. The contribution of methanogenesis from methylated compounds to terrestrial CH_4_ emissions is considered to be small [9]. However, recent research suggests it to be more important [10, 13, 81, 82]. For instance, the methylotrophic *Methanomassiliicoccales* were the second most abundant methanogenic group in Zoige peatlands [83] and also highly abundant in wetlands in northeast Germany [84].

Furthermore, we wanted to know if methanogens exhibited a differential gene expression across seasons. For this purpose, we assessed broad functional categories of mRNA transcripts taxonomically binned to Euryarchaeota. Methanogen transcript profiles had similar seasonal patterns in both soils. For instance, protein biosynthesis and transcription were upregulated in methanogens during winter (Supplementary Fig. 9). The upregulation of the protein biosynthesis machinery in soil microbiomes was recently attributed to diminished enzymatic reaction rates of metabolic enzymes at colder temperatures [85]. Likewise, our results point to a temperature-dependent regulation of central cellular processes in the here studied methanogens.

### High spatio-temporal dynamics of methanotrophs

**Fig. 5:**
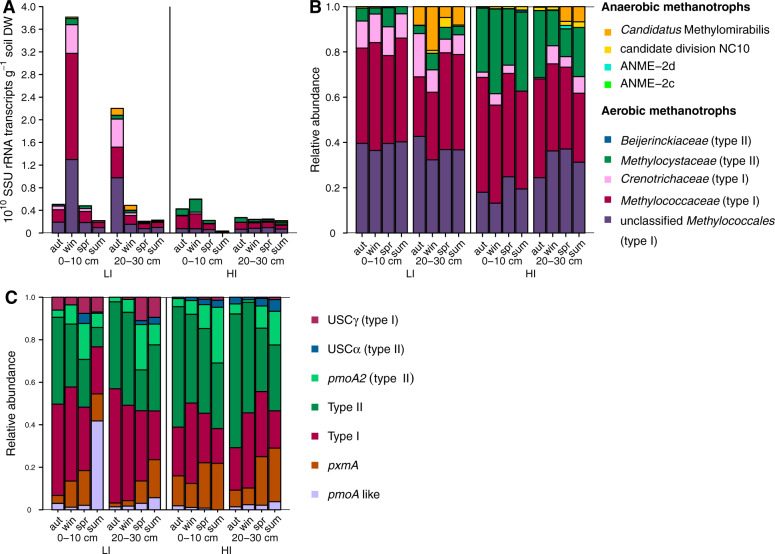
Absolute and relative methanotroph SSU rRNA abundances and composition of *pmoA* transcripts. Absolute abundances (SSU rRNA transcripts g^−1^ soil DW) of methanotrophic microorganisms (Archaea and Bacteria) (**A**), proportion of SSU rRNA transcripts belonging to methanotrophic microorganisms normalized to the total amount of SSU rRNA transcripts belonging to methanogenic Archaea and methanotrophs (**B**), and the proportion of *pmoA* groups normalized to the total amount of *pmoA* transcripts (**C**). Columns show means per seasons and depth in soils from the upper (0–10 cm) and the deeper soil layer (20–30 cm) of the grassland sites with low (LI) and high (HI) land-use intensity taken in autumn (aut) 2017 and winter (win), spring (spr) and summer (sum) 2018. “unclassified *Methylococcales*” contain *Methylococcales* unclassified at the family level and low abundance *Methylococcales* families. “pmoA like” = unclassified *pmoA*-like sequences. Bars represent the means of three replicates. Abbreviations: DW dry weight. We refer to Supplementary Figs. 10–12 showing the absolute and relative abundances of methanotroph SSU rRNA and the *pmoA* composition in the individual samples, respectively.

The aerobic methanotrophs in the soils, assessed by SSU rRNAs, mostly belonged to canonical MOBs, i.e., *Methylococcaceae*, *Crenotrichaceae*, *Methylocystaceae* (Fig. 5A, B). They were generally higher abundant in topsoils, as compared to subsoils, except of summer (Fig. 5A). Anaerobic methanotrophic bacteria (*Ca*. Methylomirabilis) and Archaea (ANME-2d) comprised a substantial part of the methanotroph community (up to 20% of all methanotrophs in subsoil) (Fig. 5B). They were present mainly in the deeper soil layer (20–30 cm), which was likely due to their sensitivity to oxygen [86]. Across seasons, methanotroph abundance (aerobic and anaerobic) was highest in autumn and winter (Fig. 5A), resembling seasonal dynamics of methanogens.

In addition to SSU rRNA, we assessed the active MOBs using transcripts of the most widespread functional marker, the alpha subunit of the pMMO [64]. While the same clades were detected, their relative abundance was sometimes different to the SSU rRNA derived MOB profiles (Fig. 5C). For instance, Methylococcales SSU rRNA transcripts (type I) clearly dominated in LI (up to 96% of all methanotroph SSU rRNA) but comprised less than 50% of *pmoA* transcripts (Fig. 5C). Generally, type II methanotrophs were more abundant in the *pmoA* than in the SSU rRNA transcripts. Studies assessing SSU rRNA composition might generally underrepresent this group. Especially in autumn and winter, the *pmoA* transcripts were dominated by canonical MOBs that probably feed on the CH_4_ produced by the methanogens. Remarkably, the proportions of *pmoA* transcripts classified as USCα, USCγ, and *pmoA2* increased in spring and summer in both sites (Fig. 5C). These *pmoA*s are assumed to be associated with atmospheric MOBs [15, 28, 87, 88]. Their increase matched the net CH_4_ uptake of the soils in spring and summer (Fig. 1A). The relative abundance of USCα and γ *pmoA* and *pmoA2* transcripts was up to 34%. Still, other type I and type II *pmoA* sequences dominated the soils. Recently, atmospheric CH_4_ oxidation in paddy soils was attributed to canonical CH_4_ oxidizers rather than USCα and USCγ [30]. Thus, also the detected type I and type II methanotrophs might be involved in atmospheric CH_4_ oxidation in spring and summer. However, it is also possible that CH_4_ is still produced in deeper soil layers and that the canonical CH_4_ oxidizers feed on this CH_4_. To complicate matters even more, the, yet only isolate of USCα methanotrophs, *Methylocapsa gorgona*, can grow at both atmospheric and elevated CH_4_ concentrations [89].

Similar as with methanogens, we wanted to explore differences in expression of general functions of methanotrophs across seasons. Transcripts taxonomically binned to gamma and alphaproteobacterial methanotrophs showed an upregulation of protein synthesis and processing as well as transcription and RNA processing in autumn and winter (Supplementary Fig. 13). This is strikingly similar to the gene expression in methanogens, providing further support that protein biosynthesis apparatus might be larger at lower temperatures [85]. In some samples only a few mRNAs could be functionally assigned. This must be considered when interpreting these results.

### Functional transcript abundances as a proxy for soil net surface CH_4_ fluxes

We have shown above that the abundance of methanogenesis-related mRNAs was a good estimator of CH_4_ fluxes in the studied soils (Fig. 4D). We now aimed to integrate methanotroph and methanogen markers to assess if a comprehensive understanding of soil CH_4_ fluxes can be derived from quantitative metatranscriptomics.

**Fig. 6:**
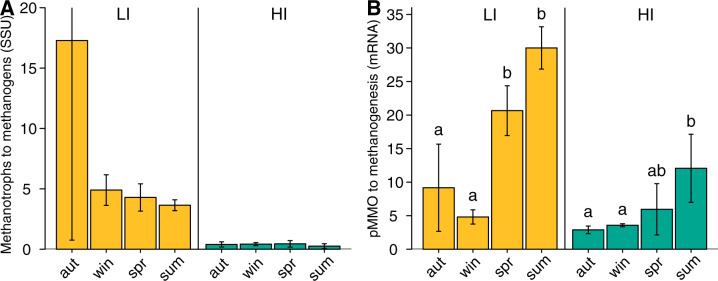
Methanotroph to methanogen ratio across seasons. The ratio of methanotroph to methanogen SSU rRNA transcripts (**A**) and pMMO to methanogenesis mRNA transcripts (**B**). The ratio was calculated with mean transcript abundances of the upper (0–10 cm) and the deeper soil layer (20–30 cm) of one soil sample. of the grassland sites with low (LI, yellow) and high (HI, turquoise) land-use intensity taken in autumn (aut) 2017 and winter (win), spring (spr) and summer (sum) 2018. Statistically significant categories of the ratios between seasons were tested with an ANOVA and subsequent post-hoc Tukey’s test at *p-adjusted* < 0.05 level.

The pMMO mRNA transcripts of both sites correlated positively with methanogenesis transcripts (*r* = 0.62, *p* < 0.001) but there was no significant correlation in SSU rRNA transcripts across sites (Supplementary Fig. 14). The correlation of mRNA transcripts suggests that methanotrophs predominantly use CH_4_ derived from methanogenesis in the soil. They thus act as a filter mitigating CH_4_ emission to the atmosphere [31]. However, there is seasonal variation; the pMMO to methanogenesis mRNA ratio was higher in spring and summer than in autumn and winter (4.8 and 3.6 in winter, and 30.0 and 12.1 in summer, in LI and HI, respectively) (Fig. 6B). Such a ratio may thus indicate whether soils are CH_4_ sources or sinks. A high methanotroph to methanogen ratio may hint at a soil being a CH_4_ sink, while a low ratio may hint at a soil being a net CH_4_ source. Yet, it is necessary to consider transcriptional activity since the ratio of methanotroph to methanogen SSU rRNA was not indicative of soils’ CH_4_ fluxes (Fig. 6A). Furthermore, the MOB community composition could be an additional indicator for soil CH_4_ uptake since a high proportion of atmospheric CH_4_ oxidizers in the *pmoA* transcripts was linked to net CH_4_ uptake of the soils.

## Conclusions

This study is, to our knowledge, the first that uses quantitative metatranscriptomics to link CH_4_ fluxes from grasslands with CH_4_-cycling microbiomes through all seasons of the year. We validated mRNA transcripts rather than SSU rRNA transcripts to be necessary for linking microbial activity to soil net surface CH_4_ fluxes in the two studied soils measured on a daily time scale. If this holds for annual rates based on temporarily highly resolved real-time data, requires more research. Still, since the abundance of mRNA of methanogenesis pathways correlated well with the net CH_4_ fluxes, it may thus be feasible to estimate soil CH_4_ fluxes using *mcr* transcript abundances when additionally considering the transcript ratio of methanotroph and methanogen key enzymes. The latter is suggested by the different ratios between the seasons in both grasslands.

Soils are the largest biological sink for atmospheric CH_4_, an important ecosystem function given the increasing concentration of atmospheric CH_4_ [1]. However, its magnitude and controlling factors are currently poorly constrained [3, 27]. Our study adds to the growing body of literature (e.g., [30]) that suggests that in soils with internal CH_4_ formation, such as the drained peatlands investigated here, many methanotroph groups contribute to atmospheric CH_4_ oxidation as compared to upland soils that are permanent net sinks of CH_4_. Stable isotope probing may be well suited to investigate this further [21–25].

We investigated 60 samples by RNAseq, a technique currently still restricted in terms of throughput and costs. Two RT qPCR studies found a relationship between *mcrA* transcript abundances and CH_4_ fluxes in a paddy soil and a peat bog, respectively [90, 91]. Parallel RT qPCRs of *mcrA* and *pmoA* transcripts might thus currently be also viable tools to estimate CH_4_ fluxes of soils from many samples and sites, respectively. Nevertheless, more large-scale studies, such as the one presented here, are encouraged to further investigate the link between methanogens and methanotrophs and CH_4_ fluxes across different soil types and seasons, especially when considering the ever decreasing costs of sequencing and further automatization in bioinformatics workflows.

## Supplementary information

Supplementary Figures

Supplementary Tables

## Data Availability

All raw sequencing data have been deposited in NCBI sequence read archive under BioProject ID PRJNA741868.
