# Supplementary material for: Linking transcriptional dynamics of CH4-cycling grassland soil microbiomes to seasonal gas fluxes
Source: ISME J. 2022 Apr 6;16(7):1788–97. doi: 10.1038/s41396-022-01229-4 (PMC9213473; doi:10.1038/s41396-022-01229-4)
Supplement: Supplementary file 1 — Supplementary Figures [file 41396_2022_1229_MOESM1_ESM.docx]

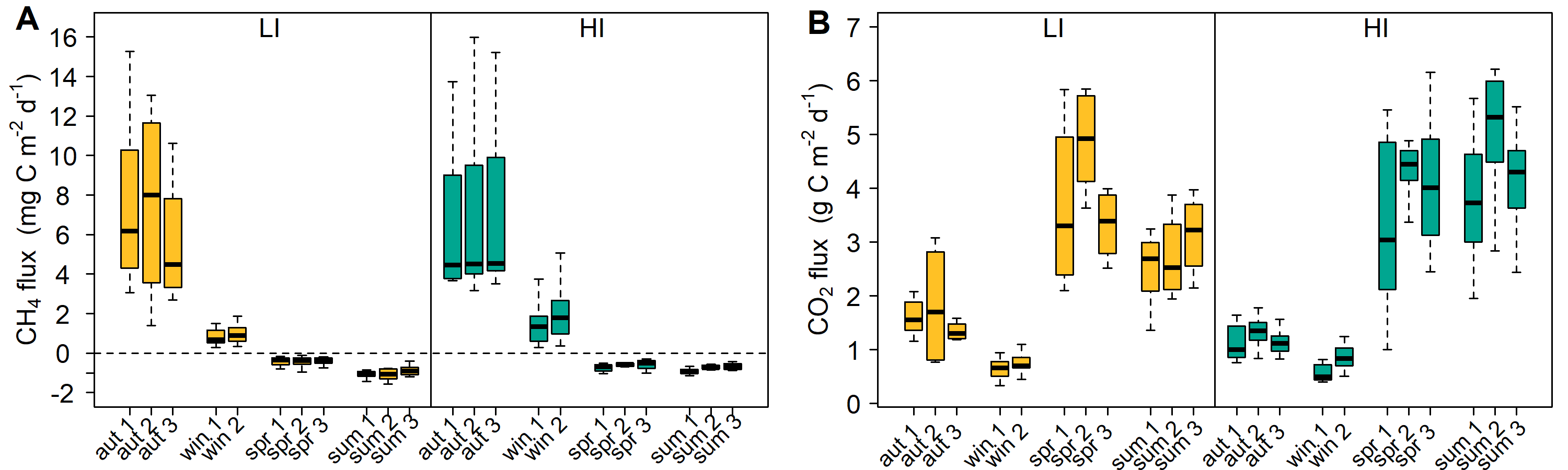


**Supplementary Figure S 1 Daily variation in Net surface gas fluxes over the year.** Gas fluxes of CH_4_ **(A)** and CO_2_ **(B)** at the grassland sites with low (yellow, LI) and high (turquoise, HI) land-use intensity in autumn (aut), winter (win), spring (spr), and summer (sum) measured in the morning (1), at noon (2) and in the evening (3). Boxes show the 25th and 75th percentiles and the lines inside the boxes indicate the median.


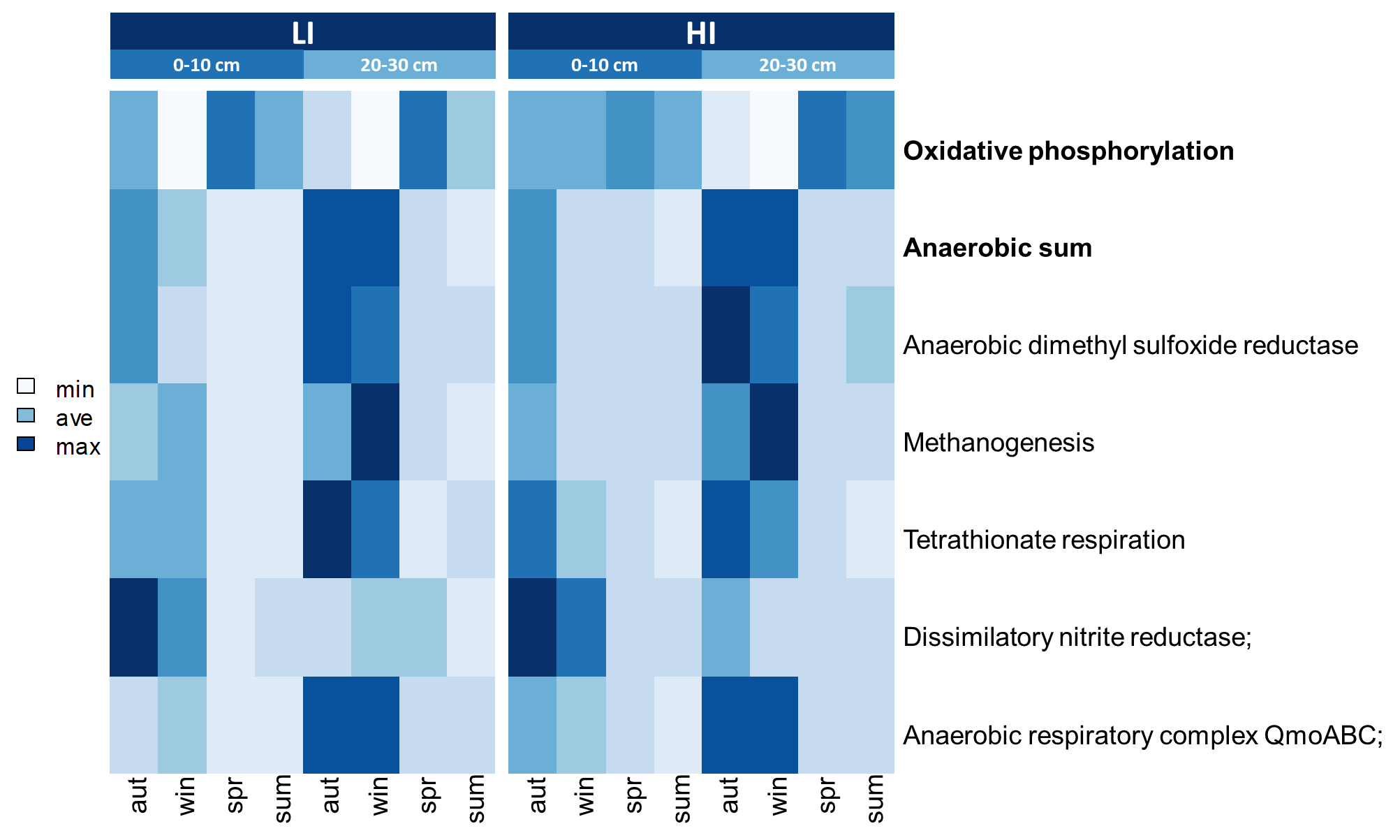


**Supplementary Figure 2 Transcription of aerobic and anaerobic function throughout the year.** Transcription of aerobic (category oxidative phosphorylation in KEGG) and anaerobic functions (sum of the following anaerobic functions in SEED: Anaerobic dimethyl sulfoxide reductase, Methanogenesis, Tetrathionate respiration, Dissimilatory nitrite reductase and Anaerobic respiratory complex QmoABC) in soils from 0-10 cm and 20-30 cm depth of two grassland sites with low (LI) and high (HI) land-use intensity in autumn (aut), winter (win), spring (spr), and summer (sum). The columns show means of three replicates per site, season, and depth. The abundance of each function was centered and scaled per site.


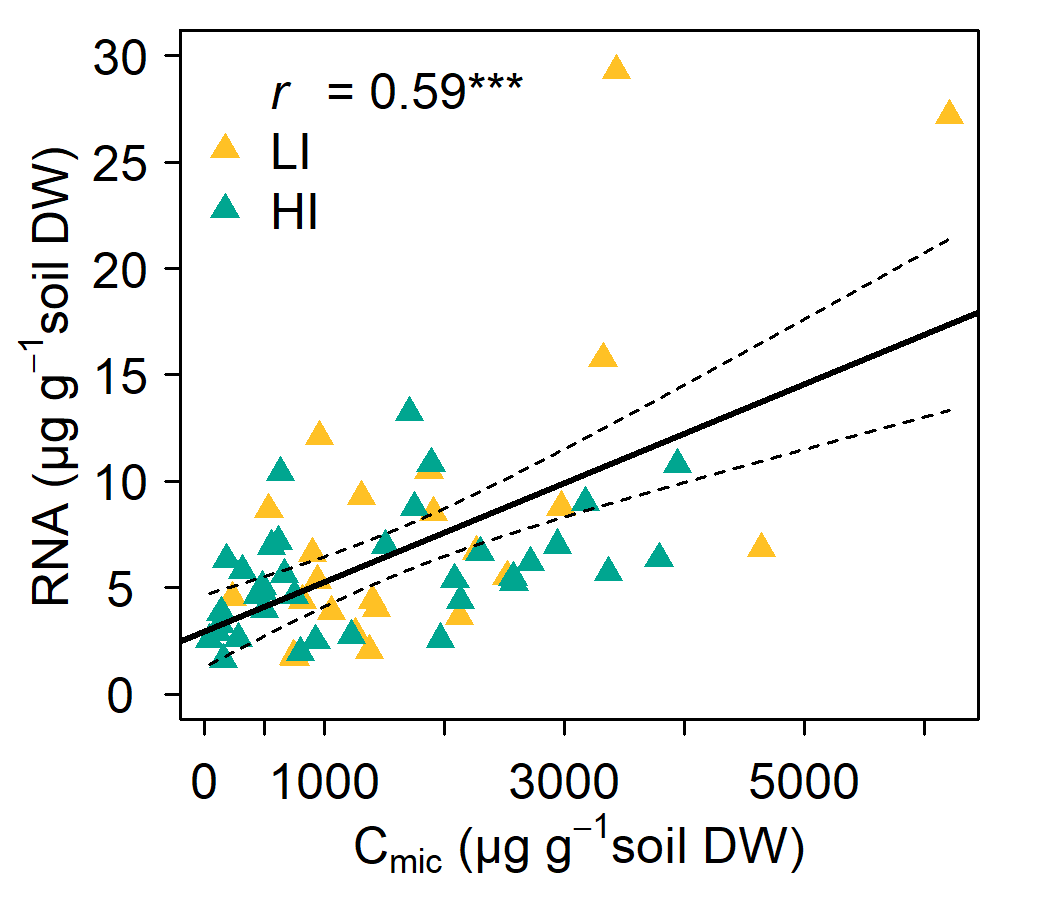


**Supplementary Figure 3 RNA and microbial carbon content.** Correlation between RNA and microbial biomass carbon content (C_mic_). Linear regression RNA = 2.970+ 0.002 C_mic_, df = 58. The “r” denotes the Pearson correlation coefficient. Significance codes: *** = p < 0.001, n= 60. LI = low land-use intensity site, HI = high land-use intensity site, DW = dry weight.


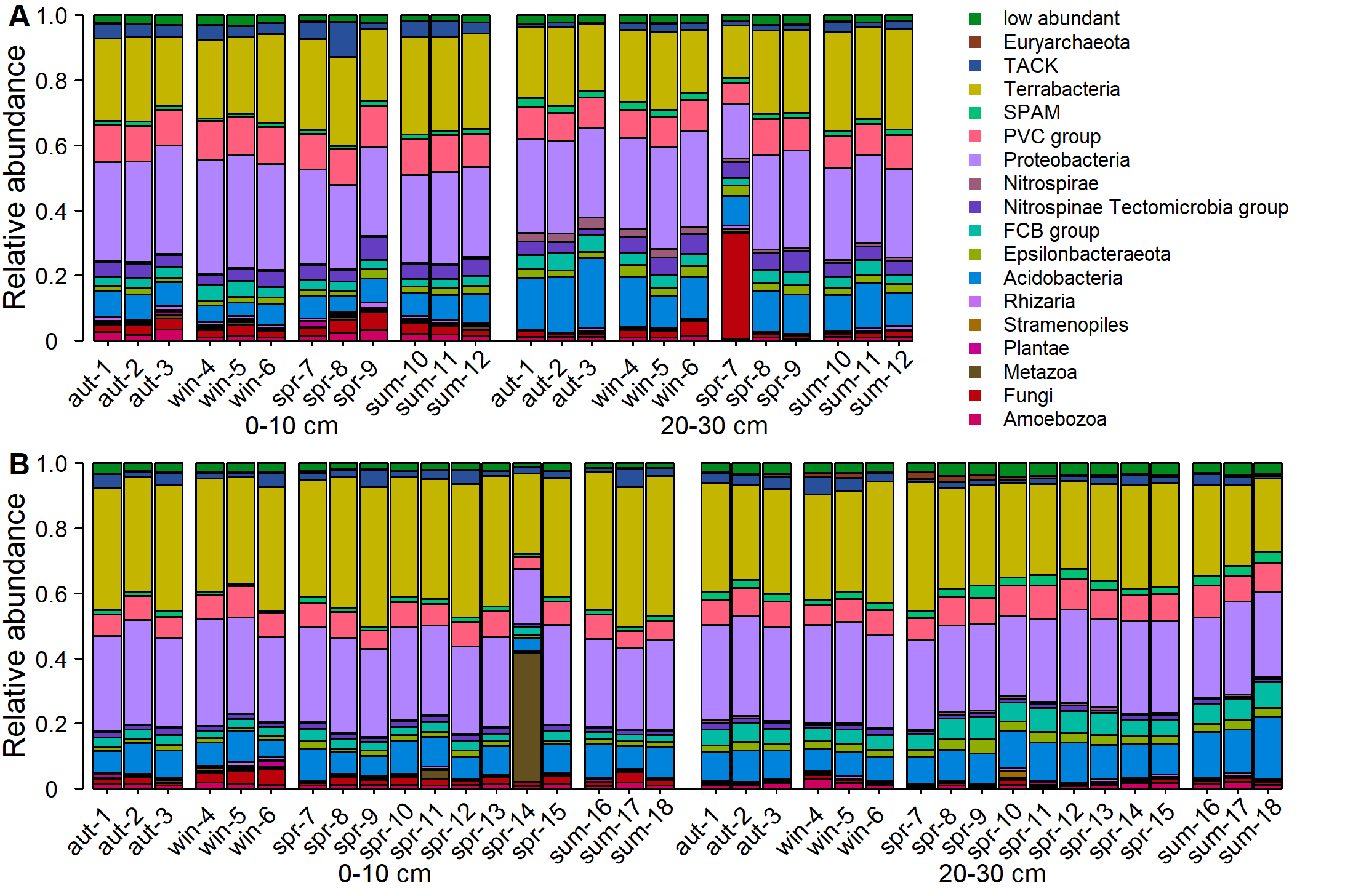


**Supplementary Figure 4 SSU rRNA community composition of prokaryotes and eukaryotes.** The proportion of transcripts belonging to eukaryotic and prokaryotic taxa normalized to the total amount of SSU rRNA transcripts. Bars show individual samples across seasons in soils from 0-10 cm and 20-30 cm depth of two grassland sites with low (LI) **(A)** and high (HI) **(B)** land-use intensity in autumn (aut), winter (win), spring (spr), and summer (sum). “low_abundant” contains taxa with abundance lower than 0.015%. The individual soil cores were numbered from 1 - 12 and 1 ‑ 18, in LI and HI, respectively. Samples with identical numbers from the upper and deeper soil layer originate from the same soil core.


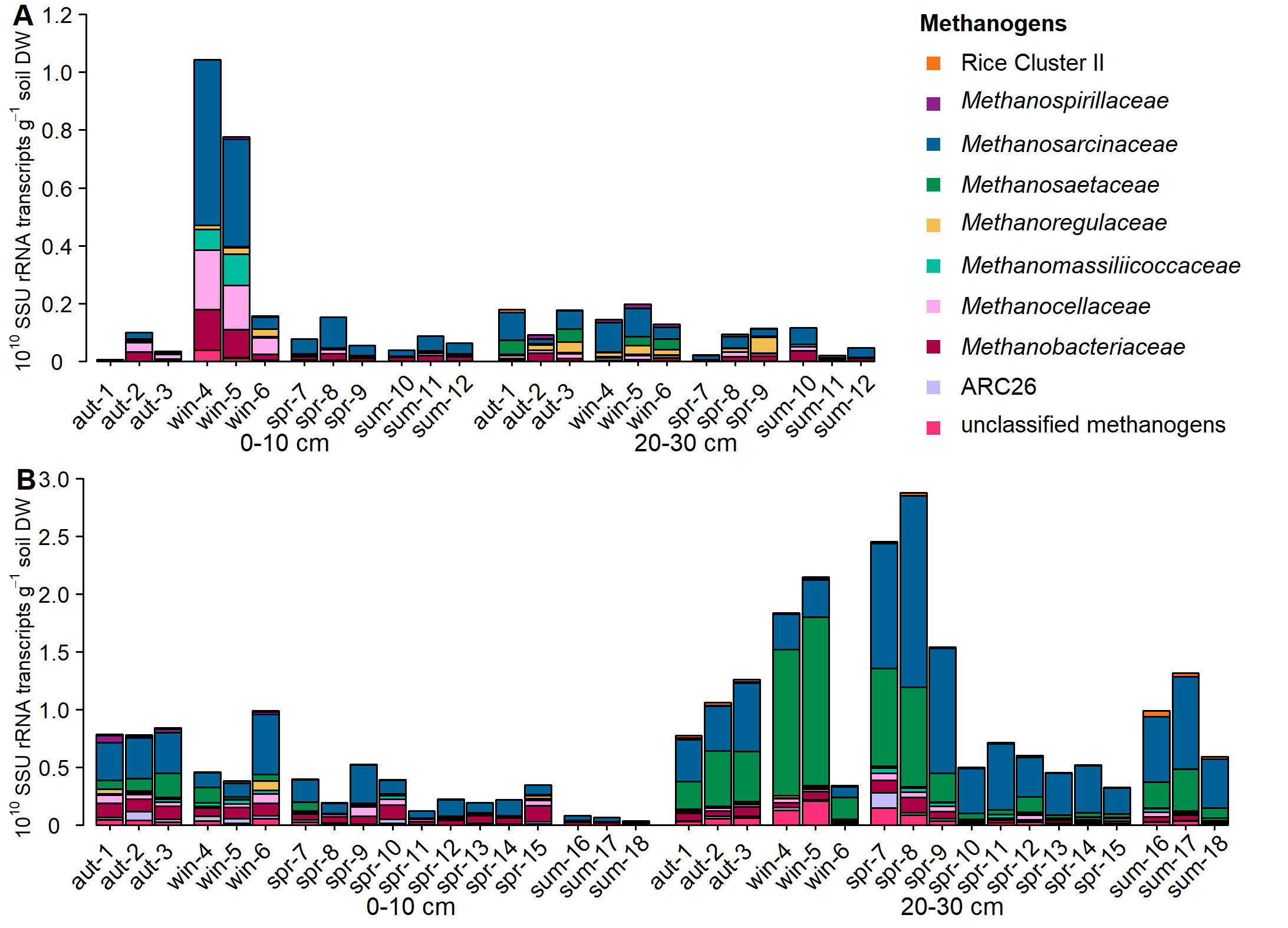


**Supplementary Figure 5 Absolute abundances of methanogen SSU rRNA across seasons and depths.** Absolute abundances (SSU rRNA transcripts g-1 soil DW) of methanogenic Archaea. Columns show individual samples in soils from 0-10 cm and 20-30 cm depth of two grassland sites with low (LI) **(A)** and high (HI) **(B)** land-use intensity taken in autumn (aut) 2017, winter (win), spring (spr), and summer (sum) 2018. “unclassified methanogens” contains methanogens unclassified at the class level and low abundance methanogenic groups. DW = dry weight. The individual soil cores were numbered from 1 - 12 and 1 ‑ 18, in LI and HI, respectively. Samples with identical numbers from the upper and deeper soil layer originate from the same soil core.


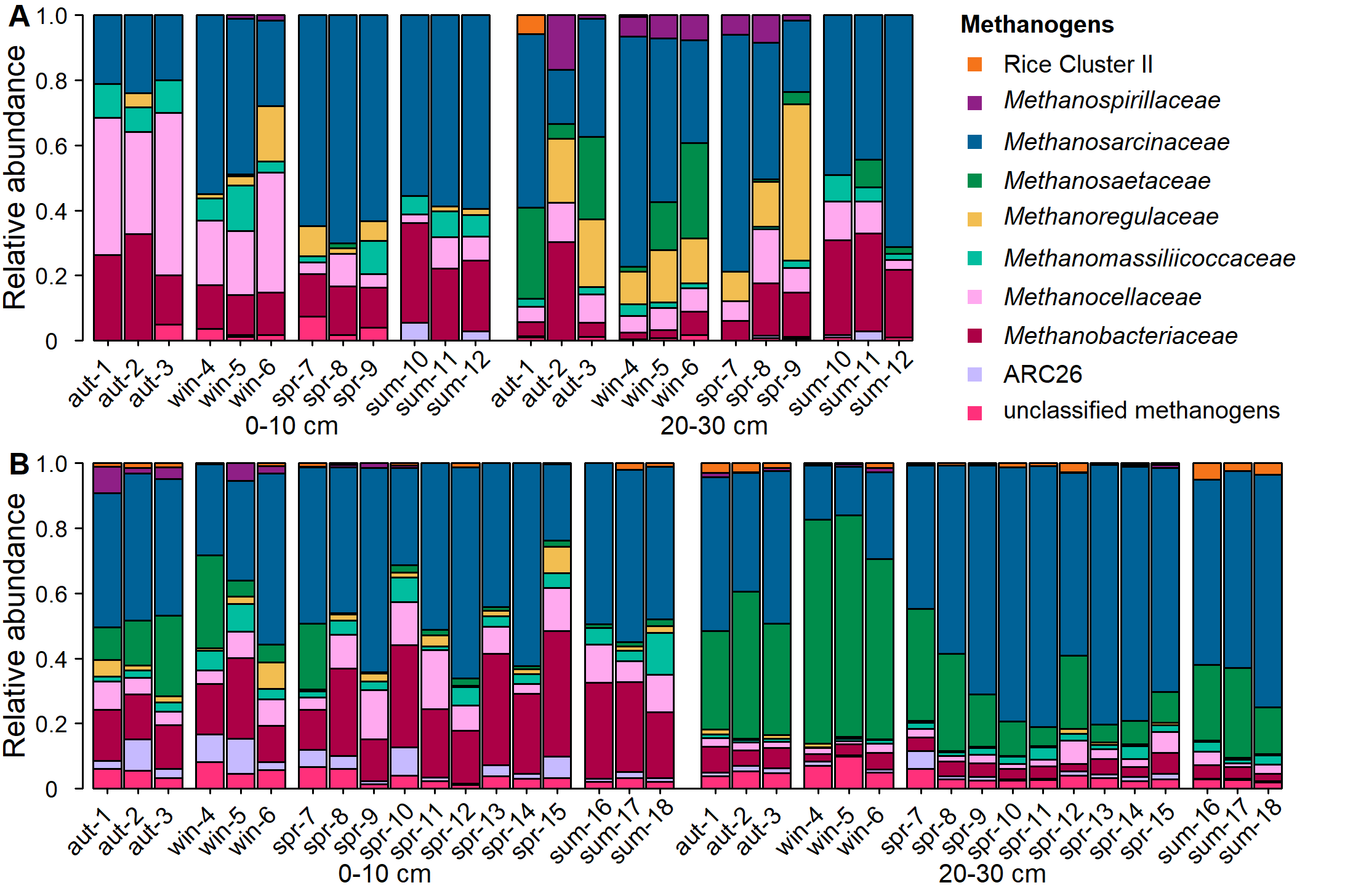


**Supplementary Figure 6** **Relative abundances of methanogen SSU rRNA across seasons and depths.** The proportions of transcripts belonging to methanogenic Archaea normalized to the total amount of transcripts belonging to methanogenic Archaea. Columns show individual samples in soils from 0-10 cm and 20-30 cm depth of two grassland sites with low (LI) **(A)** and high (HI) **(B)** land-use intensity taken in autumn (aut) 2017, winter (win), spring (spr), and summer (sum) 2018. “unclassified methanogens” contains methanogens unclassified at the class level and low abundance methanogenic groups. The individual soil cores were numbered from 1 - 12 and 1 ‑ 18, in LI and HI, respectively. Samples with identical numbers from the upper and deeper soil layer originate from the same soil core.


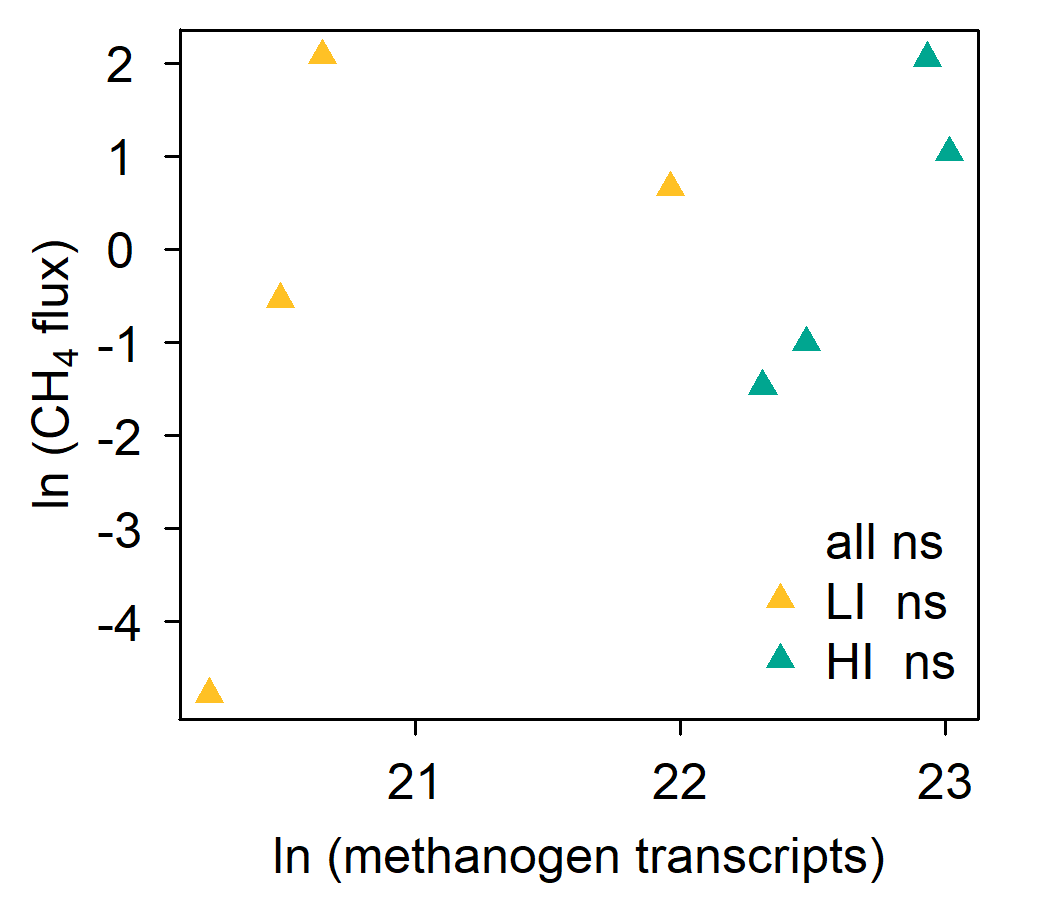


**Supplementary Figure 7 Correlation between SSU rRNA transcript abundance and CH_4_ flux.** Linear correlation of absolute abundances of methanogen SSU rRNA transcripts and CH_4_ flux. Points represent mean values per core and seasons for SSU rRNA and daily average of CH_4_ fluxes at each site, n= 8. Abbreviations: LI = low land-use intensity site, HI = high land-use intensity site, ns = not significant.


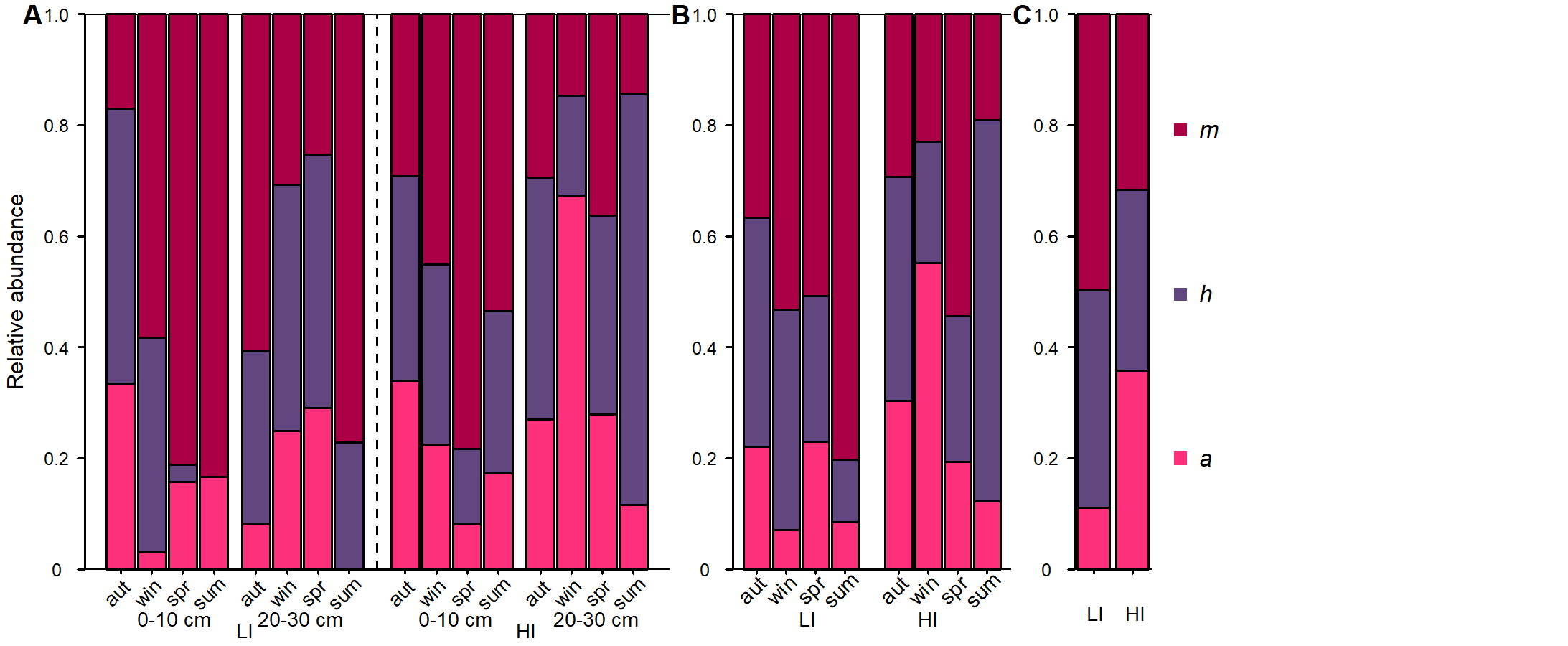


**Supplementary Figure 8 Share of mRNA transcripts belonging to acetoclastic (a), hydrogenotrophic (h), and methylotrophic (m) pathways.** Proportion of methanogenesis mRNA transcripts assigned to taxonomic groups normalized to the total amount of transcripts assigned to one of these three pathways. Bars show means in soils of the upper (0-10 cm) and the deeper soil layer (20-30 cm) in the two grasslands sites with low (LI) and high (HI) land-use intensity taken in taken in autumn (aut) 2017, winter (win), spring (spr), and summer (sum) 2018 **(A)**, averaged over the two soil depths **(B)**, and as total average over soil depth and season **(C)**.


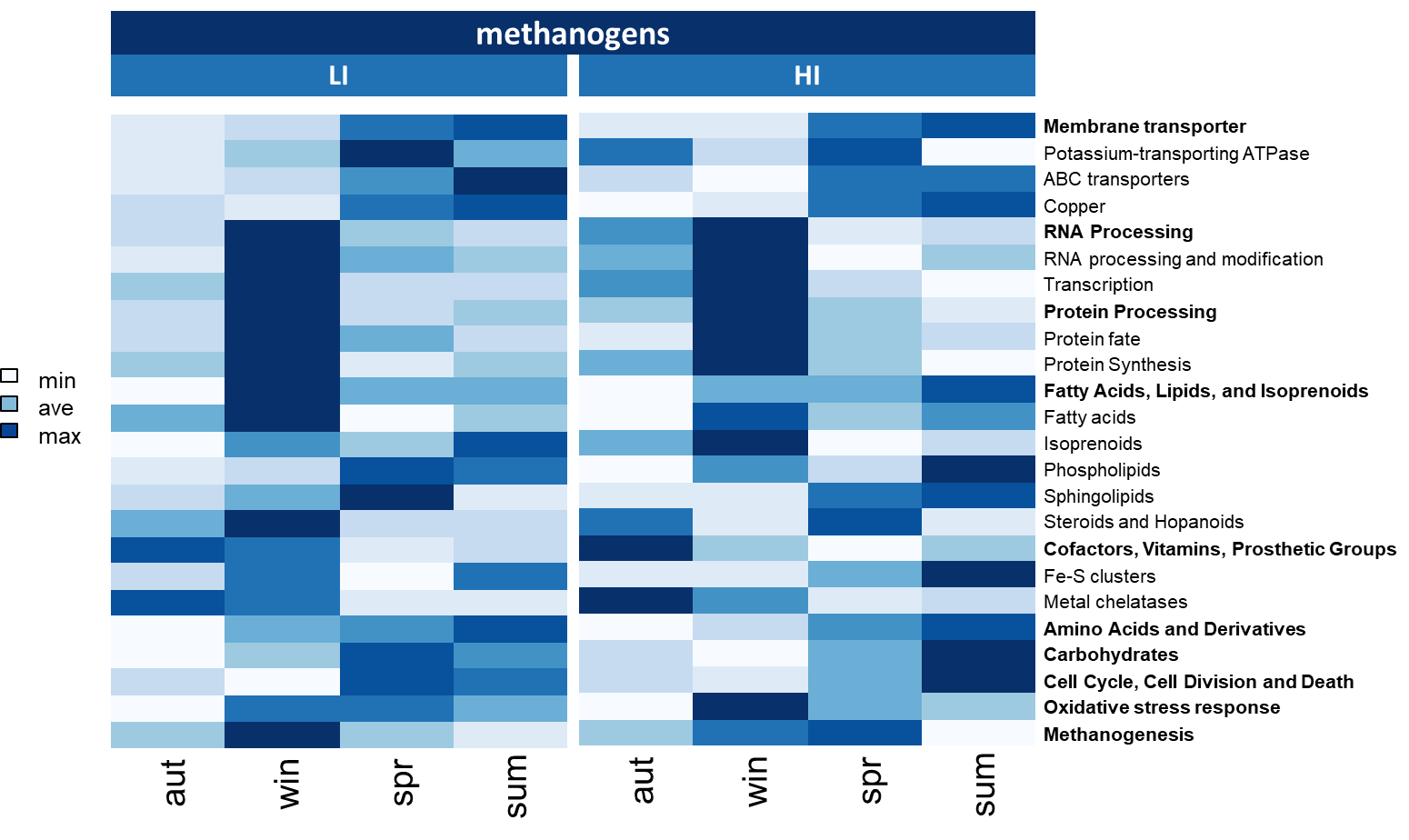


**Supplementary Figure 9 Transcriptional activity of methanogens throughout the year.** Transcription of SEED categories in methanogens in soils from 0-10 cm and 20-30 cm depth of two grassland sites with low (LI) and high (HI) land-use intensity in autumn (aut), winter (win), spring (spr), and summer (sum). The columns show means of three replicates per site, season, and depth. The abundance of each function was centered and scaled per site.


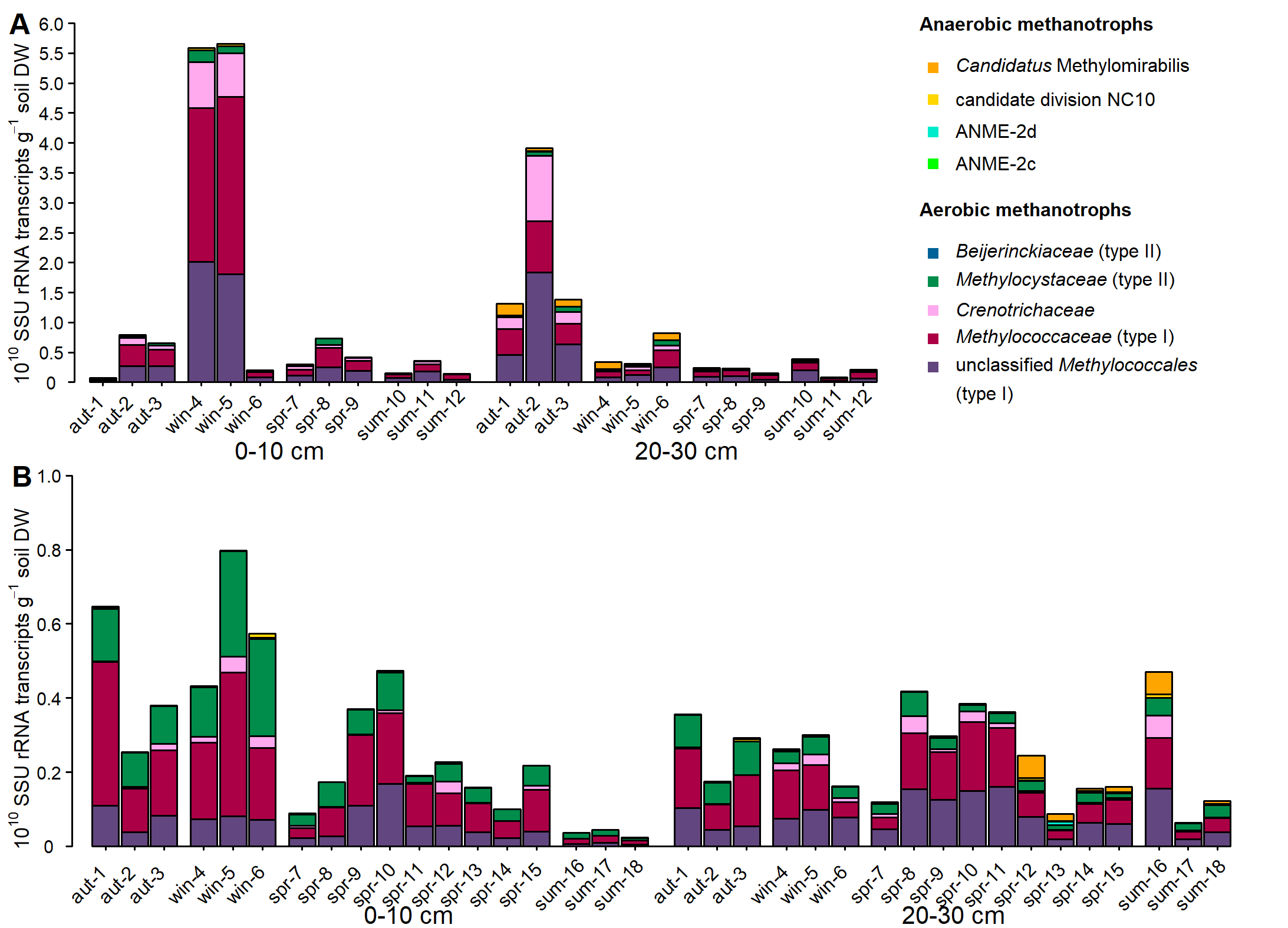


**Supplementary Figure 10 Absolute** **abundances of methanotroph SSU rRNA across seasons and depths.** Absolute abundances (SSU rRNA transcripts g-1 soil DW) of methanotrophic Bacteria and Archaea normalized to the total amount of transcripts belonging to methanogenic Archaea. Columns show individual samples in soils from 0-10 cm and 20-30 cm depth of two grassland sites with low (LI) **(A)** and high (HI) **(B)** land-use intensity taken in autumn (aut) 2017, winter (win), spring (spr), and summer (sum) 2018. “unclassified Methylococcales” contains Methylococcales unclassified at the family level and low abundance Methylococcales families. DW = dry weight.


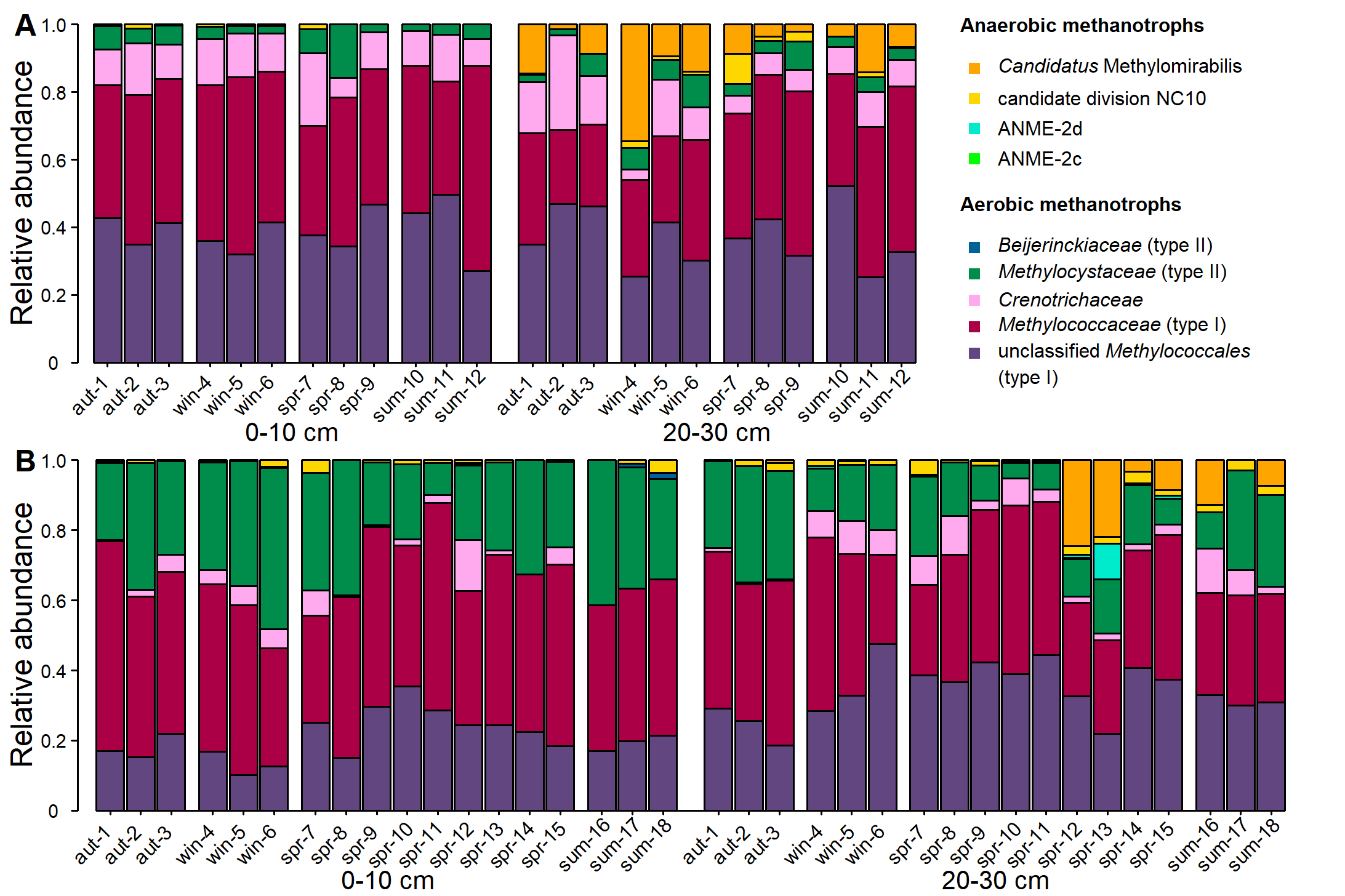


**Supplementary Figure 11 Relative abundances of methanotroph SSU rRNA across seasons and depths.** The proportion of transcripts belonging to methanotrophic Bacteria and Archaea normalized to the total amount of transcripts belonging to methanogenic Archaea. Columns show individual samples in soils from 0-10 cm and 20-30 cm depth of two grassland sites with low (LI) **(A)** and high (HI) **(B)** land-use intensity taken in autumn (aut) 2017, winter (win), spring (spr), and summer (sum) 2018. “unclassified Methylococcales” contains Methylococcales unclassified at the family level and low abundance Methylococcales families.


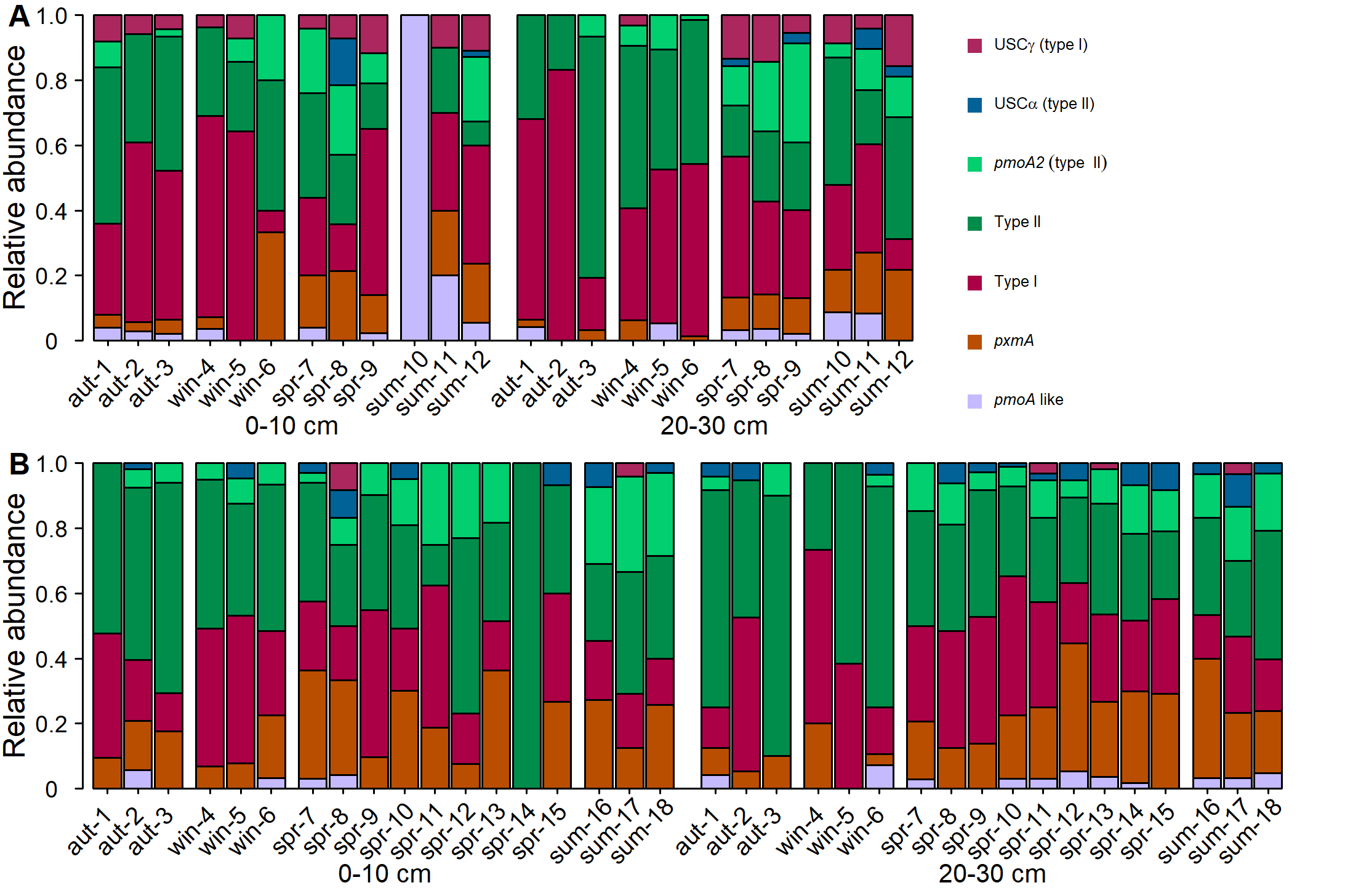


**Supplementary Figure 12 Composition of *pmoA* transcripts across seasons and depths.** The proportion of *pmoA* groups normalized to the total amount of *pmoA* transcripts. Columns show individual samples in soils from 0-10 cm and 20-30 cm depth of two grassland sites with low (LI) **(A)** and high (HI) **(B)** land-use intensity taken in autumn (aut) 2017, winter (win), spring (spr), and summer (sum) 2018. “pmoA_like” = unclassified *pmoA*-like sequences.


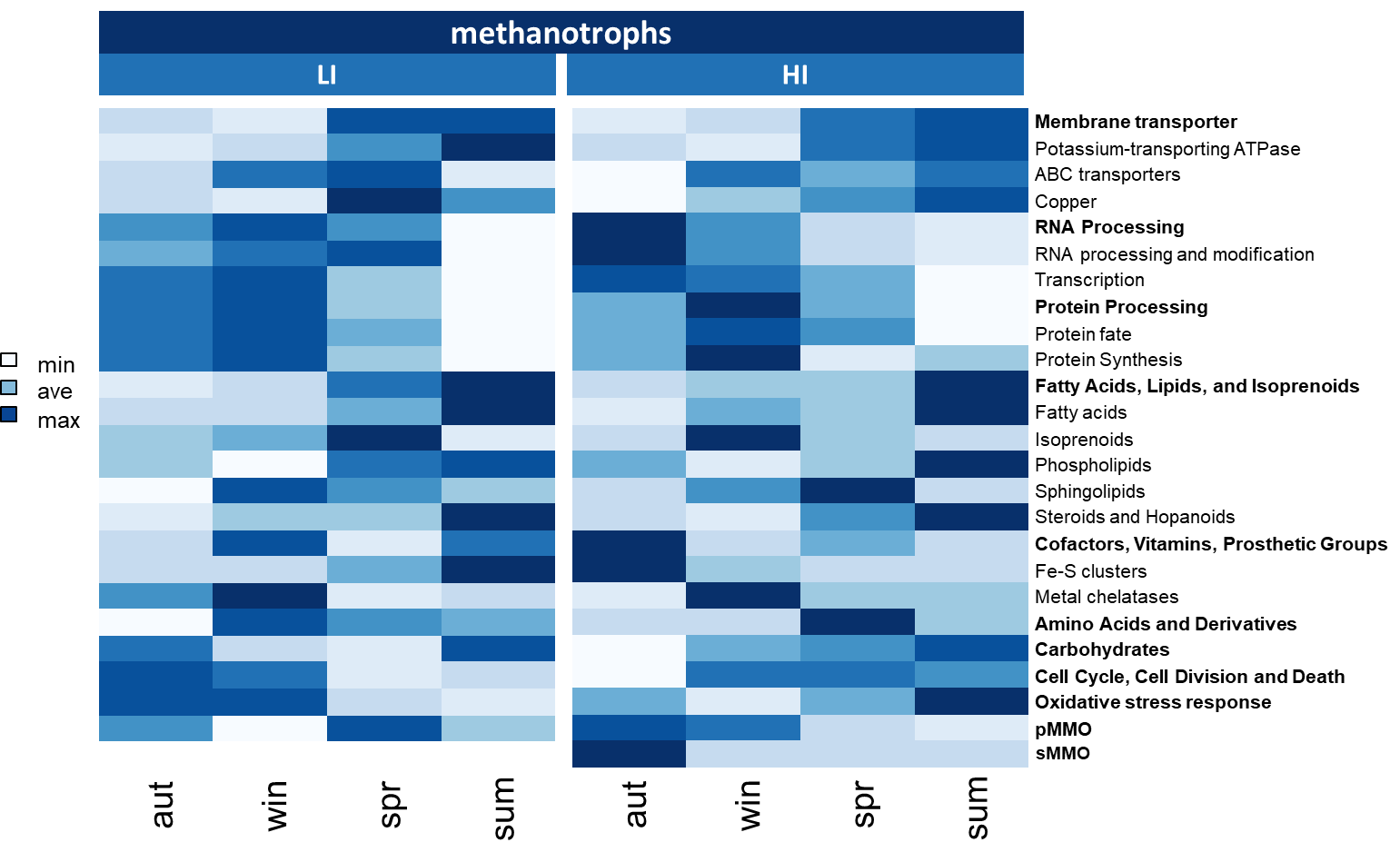


**Supplementary Figure 13 Transcriptional activity of methanotrophs throughout the year.** Transcription of SEED categories in methanotrophs in soils from 0-10 cm and 20-30 cm depth of two grassland sites with low (LI) and high (HI) land-use intensity in autumn (aut), winter (win), spring (spr), and summer (sum). The columns show means of three replicates per site, season, and depth. The abundance of each function was centered and scaled per site.


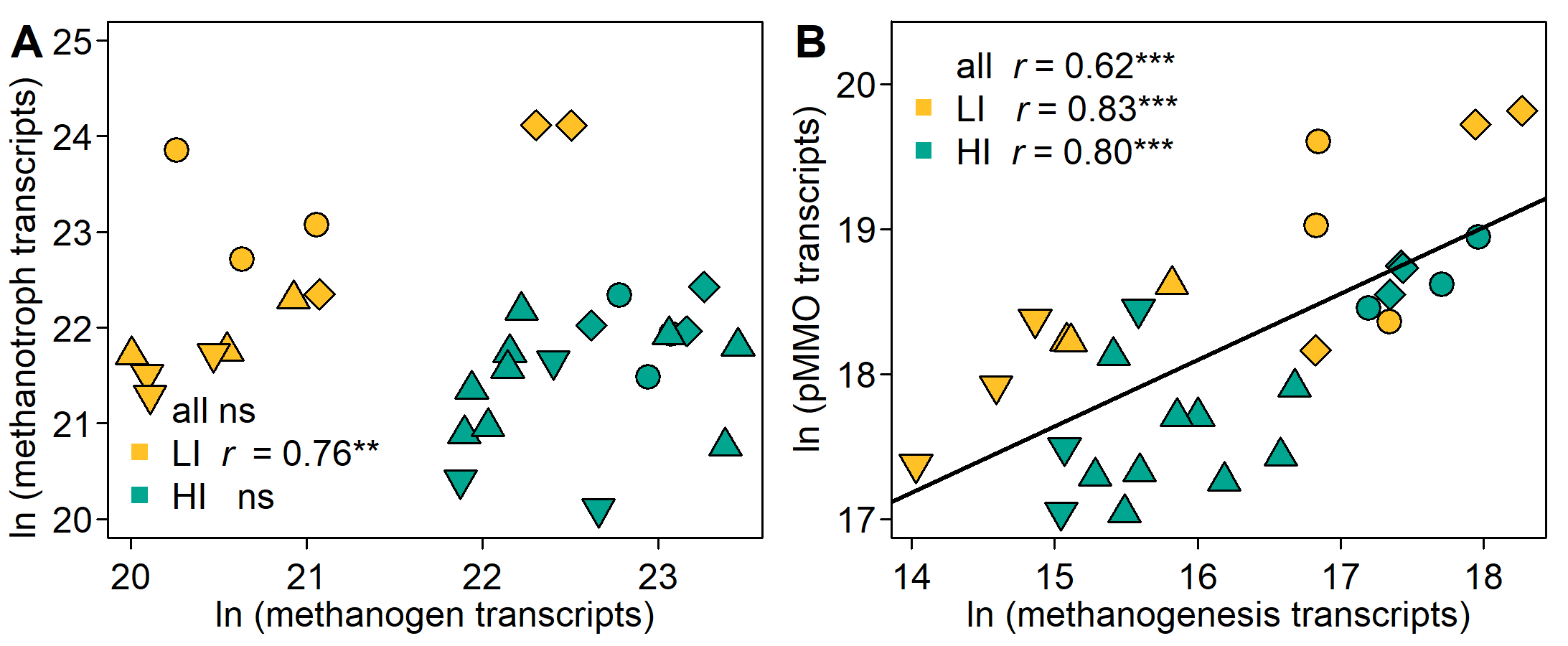


**Supplementary Figure 14 Correlation between methanogens and methanotrophs.** Linear correlation of absolute abundances of **(A)** methanogen with methanotroph SSU rRNA transcripts and **(B)** pMMO with methanogenesis mRNA transcripts in the soil cores, n = 30. Points represent mean values per core (means of both depths). The different colors represent the site with low (LI, yellow) and high (HI, turquoise) land-use intensity. Samples from autumn, winter, spring, and summer are depicted as circles, diamonds, upward-pointing triangles, and downward-pointing triangles, respectively. Significance codes: ** = *p* < 0.01, *** = *p* < 0.001, ns = not significant..
