# Supplementary material for: Linking transcriptional dynamics of CH4-cycling grassland soil microbiomes to seasonal gas fluxes
Source: ISME J. 2022 Apr 6;16(7):1788–97. doi: 10.1038/s41396-022-01229-4 (PMC9213473; doi:10.1038/s41396-022-01229-4)
Supplement: Supplementary file 2 — Supplementary Tables [file 41396_2022_1229_MOESM2_ESM.docx]

Table S1 Site specific soil properties (measured in the upper 10 cm).

|  | unit | LI | HI | reference |
| --- | --- | --- | --- | --- |
| coordinates |  | 53.109265 N  14.008877 E | 53.098521 N  13.611782 E | - |
| soil type |  | histosol | histosol | (1) |
| clay content | g kg^-1^ soil | 191 | 171 | (2) |
| silt content | g kg^-1^ soil | 593 | 143 | (2) |
| sand content | g kg^-1^ soil | 216 | 686 | (2) |
| total carbon | g kg^-1^ soil | 167.96 | 174.98 | (2) |
| organic carbon | g kg^-1^ soil | 108.26 | 171.06 | (2) |
| total nitrogen | g kg^-1^ soil | 16.6 | 17.1 | (2) |
| pH |  | 7.5 | 6.6 | (2) |
| bulk density | g cm^-3^ | 0.58 | 0.47 | (2) |
| grazing | livestock units * grazed days ha^-1^y^-1^ | - | 400-700 | (3) |
| mowing | cuts per year | 1-2 | sometimes 1 | (3) |

References:

1. Fischer M, Bossdorf O, Gockel S, Hänsel F, Hemp A, Hessenmöller D, et al. Implementing large-scale and long-term functional biodiversity research: The Biodiversity Exploratories. Basic Appl Ecol. 2010;11(6):473–85.

2. Täumer J, Kolb S, Boeddinghaus RS, Wang H, Schöning I, Schrumpf M, et al. Divergent drivers of the microbial methane sink in temperate forest and grassland soils. Glob Chang Biol. 2021;27(4):929–40.

3. Vogt J, Klaus V, Both S, Fürstenau C, Gockel S, Gossner M, et al. Eleven years’ data of grassland management in Germany. Biodivers Data J. 2019;7.

Table S2 Environmental parameters across seasons and depths. Means and standard deviations of temperature, redox potential, pH, ammonium (NH_4_^+^), nitrate (NO_3_^-^), microbial carbon (C_mic_) and microbial nitrogen (N_mic_) across seasons and depths.

| plot | depth | season | water content | air temp. | soil temp. | redox | pH | NH_4_^+^ | NO_3_^-^ | C_mic_ | N_mic_ |
| --- | --- | --- | --- | --- | --- | --- | --- | --- | --- | --- | --- |
|  |  |  | g H_2_0 g^-1^ soil DW | °C | °C | mV |  | µg N g^-1^ | µg N g^-1^ | µg g^-1^ | µg g^-1^ |
| SEG15 | 0-10 cm | aut | 1.17 ±0.09 | 6.1 | 7.75 | n.m.. | 7.6 ±0.1 | 38.3 ±22.1 | 3.3 ±1 | 1520.9 ±307.4 | 235.5 ± 25.6 |
| (LI) |  | win | 2.18 ±0.22 | 1.8 | -0.15 | n.m. | 7.4 ±0.1 | 64.9 ±69.8 | 1.6 ±0.4 | 4055.4 ±1920.5 | 641.7 ± 294.8 |
|  |  | spr | 0.91 ± 0.04 | 22.0 | 18.24 | 296 ± 22 | 7.5 ±0.1 | 49.4 ±6.3 | 8.2 ±1.4 | 3647.6 ±882.3 | 529.8 ± 63.7 |
|  |  | sum | 0.6 ± 0.11 | 13.7 | 15.23 | 344 ± 14 | 7.2 ±0 | 65.6 ±32.3 | 12.7 ±11 | 2100.4 ±178.2 | 343.4 ± 54.8 |
|  | 20-30 cm | aut | 1.96 ± 0.03 | 6.1 | 8.55 | n.m. | 7.7 ±0 | 28.5 ±26.6 | bdl | 812.6 ±238.1 | 144.7 ± 9 |
|  |  | win | 1.16 ±0.15 | 1.8 | 0.68 | -208 ± 26 | 7.6 ±0 | 11 ±3.9 | 1.2 ±0.2 | 602.9 ±319.3 | 121.7 ± 51.7 |
|  |  | spr | 0.88 ±0.03 | 22.0 | 15.25 | 125 ± 93 | 7.8 ±0 | 7 ±2.5 | 6.6 ±2.3 | 1297.7 ±208.5 | 189.9 ± 39.3 |
|  |  | sum | 0.68 ±0.03 | 13.7 | 15.42 | 302 ± 22 | 7.4 ±0 | 23.7 ±19.8 | 1.7 ±1.3 | 968.2 ±263.7 | 174.2 ± 49.6 |
| SEG9 | 0-10 cm | aut | 1.21 ±0.12 | 5.9 | 8.4 | n.m. | 6.8 ±0.1 | 34.2 ±10.7 | 1.1 ±0.4 | 2120.3 ±522.7 | 304.5 ± 64.2 |
| (HI) |  | win | 1.56 ±0.16 | 2.6 | 0.82 | n.m. | 6.7 ±0.2 | 50.8 ±16.6 | 1 ±0.8 | 3148.4 ±1246.2 | 440.3 ± 87.3 |
|  |  | spr | 0.61 ±0.09 | 23.6 | 18.91 | 268 ± 63 | 6.8 ±0.1 | 30.4 ±26 | 30 ±36.2 | 2336.4 ±786.5 | 314.7 ± 105.7 |
|  |  | sum | 0.48 ±0.09 | 13.8 | 17.69 | 334± 8 | 6.3 ±0.1 | 58.2 ±13.2 | 54.8±54.4 | 1923.6 ±673.3 | 262.1 ± 121.4 |
|  | 20-30 cm | aut | 1.2 ±0.21 | 5.9 | 9.43 | n.m. | 6.9 ±0.1 | 14.5 1±2.8 | 1.2 ±0.3 | 456.1 ±233.1 | 116.8 ±28.7 |
|  |  | Win | 1.44 ±0.59 | 2.6 | 2.21 | -160 ± 94 | 6.7 ±0.1 | 20.9 ±14.1 | 2.3 ±3.3 | 533.5 ±244.7 | 112.5 ±26.8 |
|  |  | Spr | 0.9 ±0.26 | 23.6 | 15.3 | 264 ±14 | 6.7 ±0.1 | 3.9 ±1.9 | 0.7 ±0.7 | 284.1 ±231.9 | 68.6 ±35 |
|  |  | Sum | 1.52 ±0.13 | 13.8 | 16.19 | 326 ± 20 | 6.4 ±0.1 | 20.9 ±4.8 | 15.2 ±6.9 | 602.5 ±89.3 | 130.3 ±15 |

Abbreviation: temp. = temperature, n.m. = not measured

Table S3 Relative abundances of all taxa and relative and absolute abundances of CH_4_-cycling taxa in the individual samples across seasons and depths in the grassland site with low land-use intensity (LI) in the SSU rRNA sequences.

|  |  | **all taxa** | | | **CH_4_-cycling taxa** | | | | | | | |
| --- | --- | --- | --- | --- | --- | --- | --- | --- | --- | --- | --- | --- |
|  |  | **rel abundance [%]** | | | **rel abundance [%]** | | | | **abs abundance [*10^8^ trancripts g^-1^ DW]** | | | |
| **season** | **depth** | **arc** | **bac** | **euk** | **gens** | **trophs** | **NC10** | **ANME** | **gens** | **trophs** | **NC10** | **ANME** |
| aut | 10 | 4.30 | 82.60 | 8.45 | 0.01 | 0.11 | 0.00 | 0.00 | 0.6 | 7.5 | 0.0 | 0.00 |
| aut | 30 | 0.71 | 92.21 | 4.34 | 0.05 | 0.33 | 0.03 | 0.00 | 17.7 | 126.3 | 12.0 | 0.00 |
| aut | 10 | 3.75 | 86.65 | 7.31 | 0.03 | 0.27 | 0.00 | 0.00 | 10.0 | 77.7 | 1.0 | 0.00 |
| aut | 30 | 1.31 | 94.28 | 3.85 | 0.11 | 0.66 | 0.12 | 0.00 | 17.9 | 111.4 | 19.6 | 0.00 |
| aut | 10 | 3.49 | 78.75 | 11.77 | 0.01 | 0.19 | 0.00 | 0.00 | 3.4 | 65.3 | 0.2 | 0.00 |
| aut | 30 | 1.46 | 93.18 | 2.98 | 0.03 | 1.39 | 0.02 | 0.00 | 9.2 | 385.7 | 5.6 | 0.00 |
| win | 10 | 4.46 | 85.27 | 6.77 | 0.11 | 0.59 | 0.00 | 0.00 | 104.4 | 554.7 | 4.2 | 0.00 |
| win | 30 | 2.13 | 92.08 | 4.46 | 0.10 | 0.15 | 0.09 | 0.00 | 14.4 | 21.7 | 12.4 | 0.00 |
| win | 10 | 3.30 | 85.17 | 9.10 | 0.09 | 0.66 | 0.00 | 0.00 | 77.6 | 562.0 | 3.5 | 0.00 |
| win | 30 | 2.60 | 90.78 | 4.31 | 0.36 | 0.49 | 0.06 | 0.00 | 19.8 | 27.4 | 3.2 | 0.00 |
| win | 10 | 3.28 | 87.74 | 5.53 | 0.09 | 0.11 | 0.00 | 0.00 | 15.6 | 19.6 | 0.1 | 0.00 |
| win | 30 | 2.09 | 88.25 | 7.34 | 0.09 | 0.50 | 0.09 | 0.00 | 12.8 | 69.8 | 12.2 | 0.00 |
| spr | 10 | 4.81 | 81.87 | 7.05 | 0.03 | 0.10 | 0.00 | 0.00 | 7.6 | 29.2 | 0.4 | 0.00 |
| spr | 30 | 1.22 | 61.24 | 34.49 | 0.02 | 0.16 | 0.03 | 0.00 | 2.1 | 20.1 | 4.3 | 0.00 |
| spr | 10 | 10.12 | 77.08 | 9.96 | 0.03 | 0.15 | 0.00 | 0.00 | 15.2 | 73.6 | 0.0 | 0.00 |
| spr | 30 | 1.70 | 93.34 | 3.14 | 0.07 | 0.16 | 0.01 | 0.00 | 9.3 | 22.0 | 1.1 | 0.00 |
| spr | 10 | 1.65 | 76.65 | 12.42 | 0.03 | 0.19 | 0.00 | 0.00 | 5.4 | 41.5 | 0.0 | 0.00 |
| spr | 30 | 1.55 | 95.41 | 2.34 | 0.09 | 0.11 | 0.01 | 0.00 | 11.4 | 14.0 | 0.7 | 0.00 |
| sum | 10 | 4.31 | 82.95 | 8.39 | 0.02 | 0.07 | 0.00 | 0.00 | 3.9 | 15.6 | 0.0 | 0.00 |
| sum | 30 | 2.95 | 90.96 | 3.26 | 0.06 | 0.18 | 0.01 | 0.00 | 11.7 | 37.6 | 1.4 | 0.00 |
| sum | 10 | 4.43 | 85.07 | 7.28 | 0.03 | 0.13 | 0.00 | 0.00 | 8.6 | 35.6 | 0.0 | 0.00 |
| sum | 30 | 1.48 | 92.20 | 4.20 | 0.04 | 0.13 | 0.02 | 0.00 | 2.0 | 7.2 | 1.3 | 0.00 |
| sum | 10 | 3.19 | 89.18 | 6.10 | 0.05 | 0.13 | 0.00 | 0.00 | 6.2 | 14.7 | 0.0 | 0.00 |
| sum | 30 | 2.37 | 88.83 | 4.81 | 0.05 | 0.21 | 0.02 | 0.00 | 4.6 | 19.2 | 1.5 | 0.00 |

Abbreviations: aut = autumn, win = winter, spr = spring, sum = summer, arc = archaea, bac = bacteria, euk = eukaryotes, gen = methanogens, trophs = methanotrophs, NC10 = anaerobic methanotrophs within the NC10 phylum, ANME = anaerobic methane-oxidizing Archaea, rel abundance = relative abundance (normalized to all SSU rRNA sequences).

Table S4 Relative abundances of all taxa and relative and absolute abundances of CH_4_-cycling taxa in the individual samples across seasons and depths in the grassland site with high land-use intensity (HI) in the SSU rRNA sequences.

|  |  | **all taxa** | | | **CH_4_-cycling taxa** | | | | | | | |
| --- | --- | --- | --- | --- | --- | --- | --- | --- | --- | --- | --- | --- |
|  |  | **rel abundance [%]** | | | **rel abundance [%]** | | | | **abs abundance [*10^8^ trancripts g^-1^ DW]** | | | |
| **season** | **depth** | **arc** | **bac** | **euk** | **gens** | **trophs** | **NC10** | **ANME** | **gens** | **trophs** | **NC10** | **ANME** |
| aut | 10 | 4.38 | 88.07 | 5.53 | 0.23 | 0.19 | 0.00 | 0.00 | 78.7 | 64.3 | 0.3 | 0.00 |
| aut | 30 | 3.08 | 92.67 | 2.63 | 0.35 | 0.16 | 0.00 | 0.00 | 77.6 | 35.5 | 0.1 | 0.00 |
| aut | 10 | 1.76 | 91.18 | 5.28 | 0.39 | 0.13 | 0.00 | 0.00 | 77.9 | 25.2 | 0.2 | 0.00 |
| aut | 30 | 3.46 | 92.77 | 2.46 | 0.52 | 0.08 | 0.00 | 0.00 | 106.3 | 17.1 | 0.3 | 0.00 |
| aut | 10 | 3.95 | 90.72 | 3.72 | 0.30 | 0.13 | 0.00 | 0.00 | 84.1 | 37.8 | 0.1 | 0.00 |
| aut | 30 | 4.33 | 90.67 | 3.38 | 0.64 | 0.14 | 0.00 | 0.00 | 125.8 | 28.3 | 0.9 | 0.00 |
| win | 10 | 1.90 | 87.27 | 7.85 | 0.22 | 0.21 | 0.00 | 0.00 | 45.8 | 42.9 | 0.3 | 0.00 |
| win | 30 | 6.31 | 85.18 | 5.97 | 0.99 | 0.14 | 0.00 | 0.00 | 183.8 | 25.5 | 0.5 | 0.19 |
| win | 10 | 1.22 | 85.13 | 9.16 | 0.12 | 0.25 | 0.00 | 0.00 | 38.5 | 79.6 | 0.2 | 0.00 |
| win | 30 | 5.47 | 88.07 | 4.35 | 1.39 | 0.19 | 0.00 | 0.00 | 214.6 | 29.6 | 0.4 | 0.00 |
| win | 10 | 4.32 | 78.96 | 10.67 | 0.23 | 0.13 | 0.00 | 0.00 | 99.1 | 56.2 | 1.1 | 0.00 |
| win | 30 | 2.88 | 93.12 | 2.51 | 0.55 | 0.26 | 0.00 | 0.00 | 34.3 | 16.0 | 0.2 | 0.00 |
| spr | 10 | 2.60 | 92.55 | 2.86 | 0.50 | 0.11 | 0.00 | 0.00 | 39.8 | 8.6 | 0.3 | 0.00 |
| spr | 30 | 2.93 | 94.54 | 1.72 | 1.99 | 0.09 | 0.00 | 0.00 | 245.4 | 11.4 | 0.5 | 0.06 |
| spr | 10 | 1.99 | 89.75 | 5.24 | 0.12 | 0.10 | 0.00 | 0.00 | 19.4 | 17.3 | 0.0 | 0.00 |
| spr | 30 | 3.80 | 92.09 | 2.63 | 1.77 | 0.26 | 0.00 | 0.00 | 287.5 | 41.6 | 0.2 | 0.00 |
| spr | 10 | 5.11 | 88.10 | 4.60 | 0.24 | 0.16 | 0.00 | 0.00 | 52.8 | 36.8 | 0.2 | 0.00 |
| spr | 30 | 3.21 | 93.78 | 1.66 | 1.47 | 0.28 | 0.00 | 0.00 | 154.2 | 29.2 | 0.5 | 0.00 |
| spr | 10 | 1.74 | 91.52 | 5.28 | 0.14 | 0.16 | 0.00 | 0.00 | 39.6 | 46.8 | 0.6 | 0.00 |
| spr | 30 | 2.03 | 90.21 | 6.39 | 0.96 | 0.73 | 0.00 | 0.00 | 49.9 | 38.2 | 0.2 | 0.00 |
| spr | 10 | 2.70 | 88.45 | 7.12 | 0.09 | 0.13 | 0.00 | 0.00 | 12.5 | 19.0 | 0.1 | 0.00 |
| spr | 30 | 2.44 | 93.39 | 2.53 | 0.77 | 0.39 | 0.00 | 0.00 | 71.3 | 35.9 | 0.2 | 0.05 |
| spr | 10 | 4.03 | 88.21 | 3.46 | 0.13 | 0.13 | 0.00 | 0.00 | 22.3 | 22.4 | 0.2 | 0.09 |
| spr | 30 | 1.93 | 95.09 | 1.92 | 0.41 | 0.12 | 0.04 | 0.00 | 60.4 | 17.6 | 6.6 | 0.30 |
| spr | 10 | 1.60 | 91.39 | 4.96 | 0.09 | 0.07 | 0.00 | 0.00 | 19.3 | 15.7 | 0.1 | 0.00 |
| spr | 30 | 2.64 | 92.30 | 3.02 | 0.54 | 0.07 | 0.02 | 0.01 | 45.3 | 5.7 | 2.1 | 0.88 |
| spr | 10 | 1.97 | 54.64 | 41.27 | 0.10 | 0.04 | 0.00 | 0.00 | 22.0 | 10.0 | 0.0 | 0.00 |
| spr | 30 | 3.18 | 90.74 | 3.90 | 0.35 | 0.10 | 0.01 | 0.00 | 51.9 | 14.4 | 1.0 | 0.07 |
| spr | 10 | 2.17 | 91.34 | 5.54 | 0.19 | 0.12 | 0.00 | 0.00 | 34.6 | 21.7 | 0.1 | 0.00 |
| spr | 30 | 2.25 | 91.47 | 4.71 | 0.42 | 0.18 | 0.02 | 0.00 | 32.8 | 14.2 | 1.6 | 0.16 |
| sum | 10 | 1.28 | 93.64 | 3.56 | 0.05 | 0.02 | 0.00 | 0.00 | 8.3 | 3.6 | 0.0 | 0.00 |
| sum | 30 | 3.35 | 91.64 | 3.53 | 0.30 | 0.12 | 0.02 | 0.00 | 98.8 | 40.1 | 7.0 | 0.00 |
| sum | 10 | 5.41 | 85.57 | 6.90 | 0.08 | 0.05 | 0.00 | 0.00 | 6.8 | 4.4 | 0.0 | 0.00 |
| sum | 30 | 2.92 | 89.19 | 5.35 | 0.73 | 0.03 | 0.00 | 0.00 | 131.8 | 6.1 | 0.2 | 0.00 |
| sum | 10 | 2.25 | 92.53 | 3.65 | 0.05 | 0.03 | 0.00 | 0.00 | 3.9 | 2.2 | 0.1 | 0.00 |
| sum | 30 | 1.28 | 93.91 | 3.34 | 0.46 | 0.09 | 0.01 | 0.00 | 59.3 | 11.0 | 1.2 | 0.00 |

Abbreviations: aut = autumn, win = winter, spr = spring, sum = summer, arc = archaea, bac = bacteria, euk = eukaryotes, gen = methanogens, trophs = methanotrophs, NC10 = anaerobic methanotrophs within the NC10 phylum, ANME = anaerobic methane-oxidizing Archaea, rel abundance = relative abundance (normalized to all SSU rRNA sequences).

Table S5 ANOVA results of the terms used in the dbRDA (when considering all taxa).

|  | df | sum of squares | variance explained | F | *p*-value |
| --- | --- | --- | --- | --- | --- |
| all variables |  | 2.7579 | 52.7% |  |  |
| site | 1 | 1.03728 | 20.0% | 21.1037 | 0.001 |
| depth | 1 | 1.01872 | 19.6% | 20.7262 | 0.001 |
| water content | 1 | 0.13803 | 2.7% | 2.8083 | 0.005 |
| season | 3 | 0.31121 | 6.0% | 2.1105 | 0.002 |
| soil temperature | 1 | 0.10226 | 2.0% | 2.0806 | 0.032 |
| NH_4_^+^ content | 1 | 0.05829 | 1.1% | 1.186 | 0.245 |
| NO_3_^-^ content | 1 | 0.07015 | 1.4% | 1.4272 | 0.133 |
| residual | 49 | 2.45757 | 47.3% |  |  |

Table S6 ANOVA results of the terms used in the dbRDA (when considering only CH_4_-cycling taxa).

|  | df | sum of squares | variance explained | F | *p*-value |
| --- | --- | --- | --- | --- | --- |
| all variables |  | 6.5442 | 36.1% |  |  |
| site | 1 | 2.5294 | 14.0% | 10.9274 | 0.001 |
| depth | 1 | 1.0353 | 5.7% | 4.4727 | 0.001 |
| water content | 1 | 0.9622 | 5.3% | 4.1567 | 0.001 |
| season | 3 | 1.1791 | 6.5% | 1.698 | 0.001 |
| soil temperature | 1 | 0.321 | 1.8% | 1.3869 | 0.079 |
| NH_4_^+^ content | 1 | 0.2513 | 1.4% | 1.0858 | 0.295 |
| NO_3_^-^ content | 1 | 0.2658 | 1.5% | 1.1482 | 0.230 |
| residual | 49 | 11.5738 | 63.9% |  |  |

Table S7 *ANOVA* results for the comparison of the effect of season (levels: “autumn”, “winter, “spring”, and “summer”) on methanogenesis mRNA transcript abundances (natural log transformed) for the grassland sites with low (LI) and high (HI) land-use intensity. Methanogenesis mRNA transcript abundances of the upper (0-10 cm) and lower (20-30 cm) were averaged per soil core by calculating the arithmetic mean.

|  |  | Df | Sum Sq | Mean Sq | F-value | Pr(>F) |  |
| --- | --- | --- | --- | --- | --- | --- | --- |
| LI | season | 3 | 19.401 | 6.467 | 25.6 | 0.00019 | *** |
|  | residuals | 8 | 2.021 | 0.253 |  |  |  |
| HI | season | 3 | 12.425 | 4.142 | 56.67 | 0.00001 | *** |
|  | residuals | 8 | 0.585 | 0.073 |  |  |  |

Table S8 Results of Post Hoc Tukey’s test for multiple comparisons of the effect of season (levels: “autumn”, “winter, “spring”, and “summer”) on methanogenesis mRNA transcript abundances (natural log transformed) for the grassland sites with low (LI) and high (HI) land-use intensity. Methanogenesis mRNA abundances of the upper (0-10 cm) and lower (20-30 cm) were averaged per soil core by calculating the arithmetic mean. Significant differences are highlighted with bold letters.

|  |  | diff | lwr | upr | p-adj |
| --- | --- | --- | --- | --- | --- |
| LI | **sp-a** | **-1.6656374** | **-2.9797653** | **-0.3515095** | **0.0153912** |
|  | **su-a** | **-2.5103173** | **-3.8244453** | **-1.1961894** | **0.0012798** |
|  | wi-a | 0.6748083 | -0.6393196 | 1.9889362 | 0.4086841 |
|  | su-sp | -0.8446799 | -2.1588079 | 0.469448 | 0.2447354 |
|  | **wi-sp** | **2.3404457** | **1.0263178** | **3.6545737** | **0.0020237** |
|  | **wi-su** | **3.1851257** | **1.8709978** | **4.4992536** | **0.0002495** |
| HI | **sp-a** | **-1.8050055** | **-2.5118376** | **-1.0981733** | **0.0001719** |
|  | **su-a** | **-2.3887456** | **-3.0955778** | **-1.6819135** | **0.0000219** |
|  | wi-a | -0.2204034 | -0.9272355 | 0.4864288 | 0.754611 |
|  | su-sp | -0.5837401 | -1.2905723 | 0.123092 | 0.1097982 |
|  | **wi-sp** | **1.5846021** | **0.87777** | **2.2914343** | **0.0004314** |
|  | **wi-su** | **2.1683423** | **1.4615101** | **2.8751744** | **0.0000451** |

Abbreviations: aut = autumn, win = winter, spr = spring, sum = summer, diff = , lwr =
